# GP38 as a vaccine target for Crimean-Congo hemorrhagic fever virus

**DOI:** 10.1038/s41541-023-00663-5

**Published:** 2023-05-20

**Authors:** Gabrielle Scher, Dennis A. Bente, Megan C. Mears, Maria N. B. Cajimat, Matthias J. Schnell

**Affiliations:** 1 Department of Microbiology and Immunology, Sidney Kimmel Medical College at Thomas Jefferson University, Philadelphia, PA USA; 2 Galveston National Laboratory, Department of Microbiology and Immunology, Institute for Human Infections and Immunity, University of Texas Medical Branch, Galveston, TX USA; 3 Department of Pathology, University of Texas Medical Branch, Galveston, TX USA; 4 Jefferson Vaccine Center, Sidney Kimmel Medical College, Thomas Jefferson University, Philadelphia, PA USA

**Keywords:** Inactivated vaccines, Viral vectors

## Abstract

Crimean-Congo Hemorrhagic Fever Virus (CCHFV) is a tick-borne virus that causes severe hemorrhagic disease in humans. There is a great need for effective vaccines and therapeutics against CCHFV for humans, as none are currently internationally approved. Recently, a monoclonal antibody against the GP38 glycoprotein protected mice against lethal CCHFV challenge. To show that GP38 is required and sufficient for protection against CCHFV, we used three inactivated rhabdoviral-based CCHFV-M vaccines, with or without GP38 in the presence or absence of the other CCHFV glycoproteins. All three vaccines elicited strong antibody responses against the respective CCHFV glycoproteins. However, only vaccines containing GP38 showed protection against CCHFV challenge in mice; vaccines without GP38 were not protective. The results of this study establish the need for GP38 in vaccines targeting CCHFV-M and demonstrate the efficacy of a CCHFV vaccine candidate based on an established vector platform.

## Introduction

Crimean-Congo Hemorrhagic Fever Virus (CCHFV) is an emerging infectious disease with an extensive global distribution spanning across areas of Africa, Asia, the Middle East, and Europe^1–5^. The wide range of endemic areas is due to the natural habitat of CCHFV’s tick vector, ticks of the *Hyalomma* genus^1–5^. Areas where this tick can survive are increasing due to anthropogenic factors such as habitat modification, thus increasing the areas where CCHFV can circulate^6,7^. CCHFV infects a wide range of mammalian hosts, yet it does not cause visible disease in these animals^1–5^. However, CCHFV can cause Crimean-Congo hemorrhagic fever (CCHF) in humans, which first presents with flu-like symptoms and progresses to bleeding, petechiae, and, in more severe cases, organ failure and death^1–5^. The case-fatality rate for CCHF is up to 40%^1–5^, and there are no licensed CCHFV-specific vaccines or treatments available for humans. Therefore, CCHFV is designated as a biosafety level 4 (BSL-4) pathogen, further highlighting the need for effective vaccines and therapeutics. Accordingly, CCHFV is classified as an NIH/NIAID Category A and World Health Organization (WHO) high-priority pathogen.

There have been a variety of vaccine strategies against CCHFV tested in animal models with varying success^8–10^. The only vaccine ever tested in humans was a whole inactivated virus vaccine propagated in mouse brains that reduced cases in Bulgaria, but requires BSL-4 laboratories for production and is administered as a four dose regimen^11^. While many other strategies have proven to be protective in animal models^8–10^, there are concerns regarding the clinical application of each candidate. A cell culture produced whole inactivated virus vaccine showed 80% protection in mice^12^ however, it requires a BSL-4 facility for production, which is dangerous and expensive. DNA vaccines using both the nucleoprotein (S) gene, glycoprotein (M) gene, or a combination of these antigens have demonstrated 100% protection in mice or *Cynomolgus macaques*^13–16^, but DNA vaccines have not been effective in humans. A nucleoside-modified mRNA vaccine using CCHFV nucleoprotein and/or glycoproteins also showed 100% protection in mice^10^. However, the study did not investigate the longevity of the immune responses elicited by the vaccine, which might be a problem based on the findings of waning humoral immune response to the Severe Acute Respiratory Syndrome Coronavirus 2 (SARS-CoV-2) mRNA vaccine^17^. Finally, both live Modified Vaccinia Ankara (MVA) and Vesicular Stomatitis virus (VSV) vaccines containing the CCHFV-M gene protected mice from CCHFV challenge^9,18^, but supporting clinical studies are pending. While live vaccine strategies can be effective, there is always a concern about the virulence (whether inherent or mutation acquired) of these vectors, especially when used in immunocompromised people, pregnant women, and children. Thus, there is still a great need for an effective and safe CCHFV vaccine strategy.

Rhabdoviruses, specifically rabies virus (RABV) and VSV, have been used as vaccine vectors for a variety of infectious diseases^19^, including CCHFV, as mentioned above^9^. These vectors have many advantages, including their small, easily manipulated genome that can stably express foreign glycoproteins^20,21^ and their well-established safety profiles^22–26^. Both vectors can be used as inactivated vaccines that will elicit immune responses against both foreign glycoproteins and the native rhabdoviral glycoproteins^22–25^ however, VSV has never been tested as a killed vector. The RABV vaccine has been shown to elicit long-lasting immunity in humans^27^, which is important for a vaccine platform. Moreover, a rabies-based vaccine against SARS-CoV-2 is currently being evaluated in humans^28^. Finally, RABV and CCHFV share many endemic regions, and thus a bivalent vaccine against both viruses would have a significant impact in the affected areas.

**Fig. 1:**
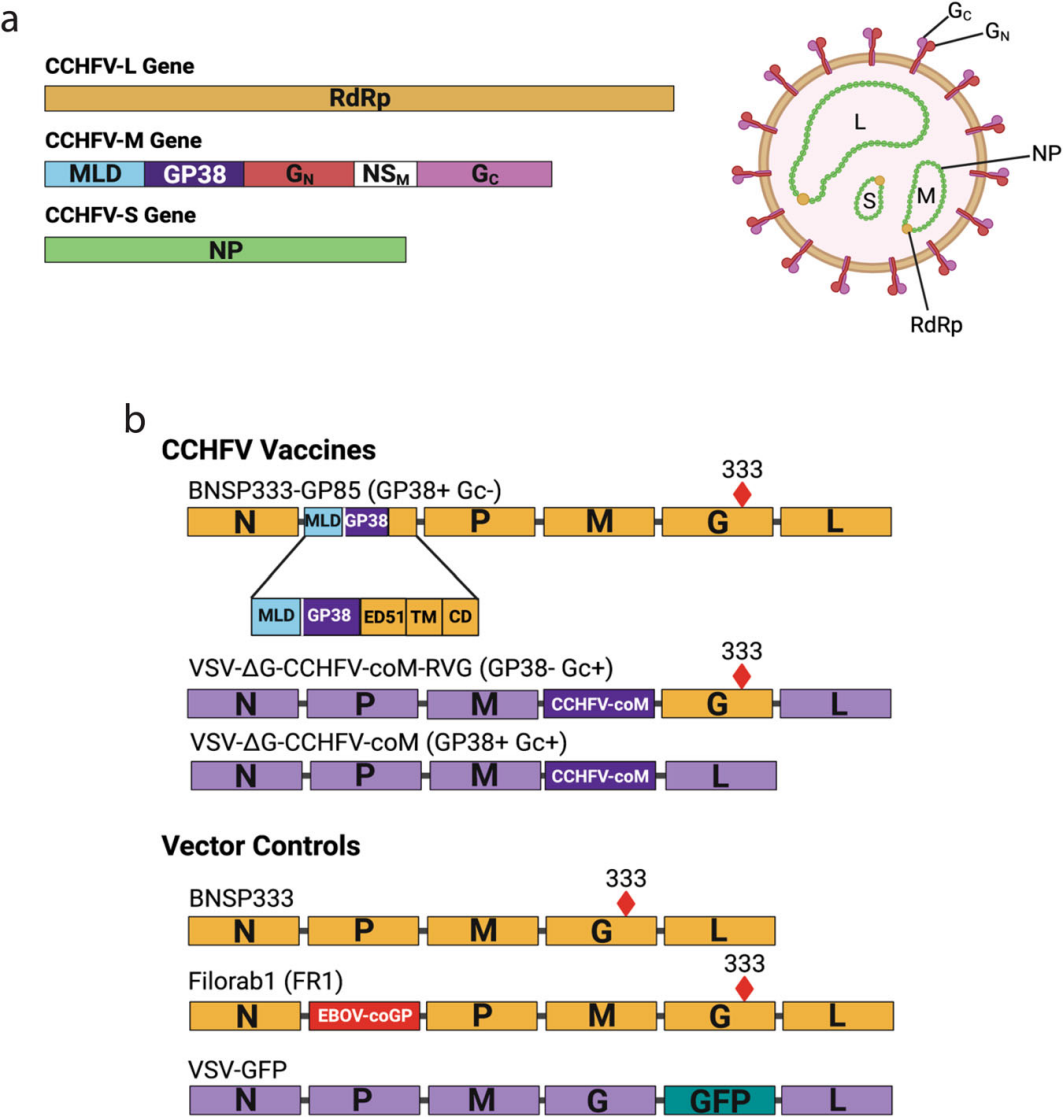
CCHFV genome and rhabdoviral-based CCHFV vaccine vector maps. Schematics of the CCHFV genome and virion (**a**), and RABV- and VSV-based CCHFV vaccines and their vector controls (**b**). All foreign genes were inserted into the BNSP333 vector between N and P and between M and L for the VSV vector. The GP85 chimeric gene is expanded to show the various sections of both GP85 and the RABV-G that were included in the gene. Attenuating R333E mutation is marked in RABV-G. RdRp RNA-dependent RNA polymerase, MLD Mucin-like domain, NS_M_ non-structural M protein, NP nucleoprotein, N nucleoprotein, P Phosphoprotein, M matrix protein, G glycoprotein, L polymerase, ED51 51 amino acids of the ectodomain, TM transmembrane domain, CD cytoplasmic domain. Created with Biorender.com.

CCHFV is a member of the order *Bunyavirales*, family *Nairoviridae*, a group of single-stranded negative-sense RNA viruses with tri-segmented genomes. Vaccine strategies targeting the CCHFV-M segment have shown protection in mouse challenge models as previously stated^9,10,13,18^. This gene encodes for the virus’s glycoproteins, specifically structural proteins G_N_ and G_C_, secreted GP38, and non-structural proteins NS_M_ and a mucin-like domain (MLD)^29^ (Fig. 1a). G_N_ and G_C_ are embedded in the membrane that encompasses the virion and mediate cell attachment and entry^29^ (Fig. 1a), and G_N_ is suspected of playing a role in virion assembly^30^. GP38 and the MLD, referred to as GP85, have been shown to play a role in the processing and trafficking of the structural glycoproteins and are indispensable for viral replication^31^. NS_M_ was shown to play a role in G_C_ processing but was not required for viral replication^31^.

Currently, there are no defined correlates of protection for CCHFV. Studies using either part of or the full-length CCHFV-M gene as a vaccine target have shown varying results regarding the vaccine’s protective efficacy^8–10^. Specifically, vaccines that induce immune responses against the full-length CCHFV-M are protective^9,18,32^, while those that only target the structural proteins are not protective against CCHFV^13,33^. The humoral immune response elicited against CCHFV-M during natural infection is specific for G_C_ and GP38^34,35^. Interestingly, although the G_C_ antibodies are neutralizing, they are not always protective^33,36,37^. GP38 antibodies are non-neutralizing, and one monoclonal antibody was shown to be protective in an adult mouse challenge model^36,37^. Additionally, a CCHFV-M based DNA vaccine study showed that GP38 was required for protection^15^. Thus, GP38 is a very attractive target antigen for a CCHFV vaccine that has not been extensively tested in the absence of other CCHFV glycoproteins.

Here, we present an effective CCHFV vaccine based on RABV virions containing membrane-anchored GP38. To demonstrate the requirement of immune responses against GP38 for protection against CCHFV, we have developed two VSV-based inactivated CCHFV vaccines containing the full M segment with or without GP38. Efficacy of this vaccine was shown in two animal models: a non-BSL-4 VSV-based surrogate challenge model for CCHFV in immunocompromised interferon α/β receptor 1 knockout (IFNAR^−/−^) mice, and challenge with wildtype CCHFV in transiently immune suppressed C57BL/6 mice. Our results indicate that immune responses against GP38 are required for protection against CCHFV and that the GP85 vaccine is an excellent candidate for a CCHFV vaccine.

## Results

### Vaccine design

**Fig. 2:**
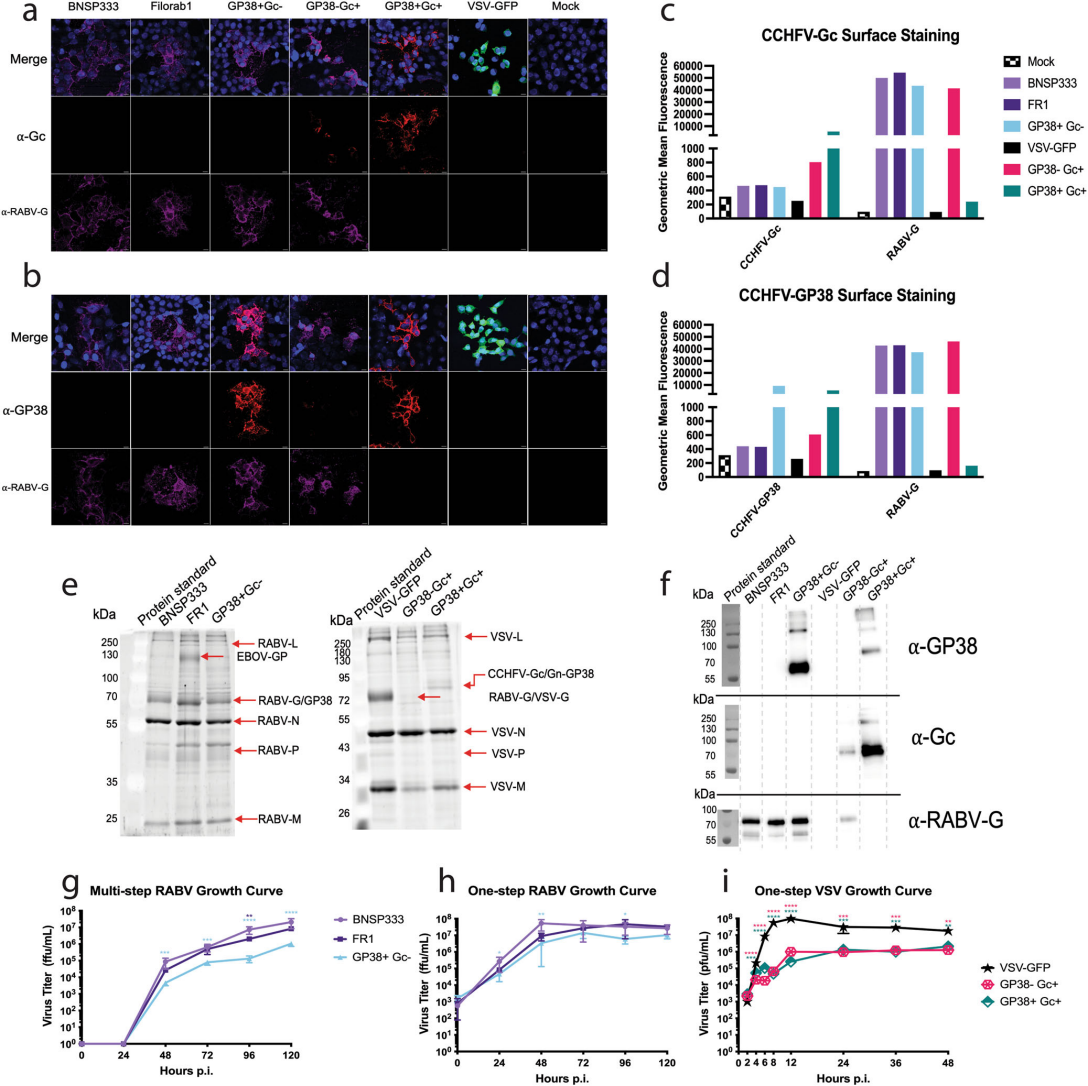
Rhabdoviral vectors express and incorporate CCHFV glycoproteins. Characterization of rhabdoviral-vectored CCHFV vaccines through Immunofluorescence (**a**, **b**), flow cytometry (**c**, **d**), SDS PAGE protein gel (**e**), Western Blot (**f**), and Growth Curves (**g**–**i**). Vero E6 cells were infected at MOI 0.01 and fixed after 72 or 24 h for RABVs and VSVs, respectively. Cells were stained with α-RABV-G 4C12 (purple) and α-CCHFV-G_C_ 11E7 (**a**) or α-CCHFV-GP38 13G8 (**b**) (red) and mounted with mounting media containing a nuclear DAPI stain (blue). In the merged images, GFP from VSV GFP is green, and areas where there is overlapping expression of RABV-G and the CCHFV glycoproteins are pink. Images were taken at 40X magnification with a 2X zoom. Scale bars represent 10 µm. **c** Vero E6 cells were infected at MOI 10 and fixed after 48 h for RABVs or infected at MOI 5 and fixed after 8 h for VSVs. Cells were probed for α-RABV-G 4C12 and α-CCHFV-G_C_ 11E7 (**c**) or α-CCHFV-GP38 13G8 (**d**) and analyzed by flow cytometry. Assay was performed multiple times, and the graph is one representative experiment. **e** SDS PAGE protein gel of sucrose purified virions. 1 µg of each virus was loaded onto the gel and all native rhabdoviral proteins and foreign proteins are indicated by the arrows next to each gel. **f** Western blot of sucrose purified virions. 1 µg of each virus was loaded onto the gel and transferred to a nitrocellulose membrane for western blotting. Blots were either probed with α-CCHFV-GP38 13G8 (top panel), α-CCHFV-G_C_ 11E7 (middle panel) or α-RABV-G 4C12 (bottom panel). **g-i** Multi-step and one-step growth curves. Cells were infected at MOI 0.01 for multi-step (**g**) or MOI 10 for one-step (**h**, **i**) growth curves and samples were titered in triplicate. Error bars represent standard deviation. Statistics are differences in titer compared to the parental vector for each growth curve (****P < 0.0001; ***P < 0.0002; **P < 0.0021; *P < 0.0332).

To construct the rhabdoviral-based CCHFV vaccines, we used the rabies vector BNSP333 and VSV vector cVSV-XN. BNSP333 is a well-characterized vector derived from RABV vaccine strain SAD-B19. SAD-B19 has been further attenuated through an arginine to glutamic acid mutation at amino acid 333 of the glycoprotein (G) gene, which reduces the vector’s neurotropism^38^ and has been used for multiple vaccine approaches (for review see^19^). cVSV-XN is based on the Indiana strain of VSV^39^, which is attenuated by an unknown mechanism. A human-codon optimized CCHFV-M (coM) gene from strain IbAr10200 was used as the antigen for these vaccines^32^. Three different CCHFV vaccines were constructed with an emphasis on GP38, which we hypothesize is required for a protective CCHFV vaccine (Fig. 1b). BNSP333-GP85 (GP38 + G_C_-) contains a modified GP85, where CCHFV GP38 is anchored in the RABV virion by the addition of 51 amino acids of the RABV glycoprotein (G) ectodomain (ED), the transmembrane domain (TM) and cytoplasmic tail (CT), as used previously to successfully incorporate other proteins into RABV virions^40–43^. Since the CCHFV MLD is cleaved and secreted during glycoprotein maturation^44^, the GP38 part is the only protein from CCHFV-M present in this vaccine (Fig. 2e, f). The second construct, VSV-ΔG-CCHFV-coM-RVG (GP38- G_C_ + ), is a VSV-vectored vaccine containing the full M gene with the terminal 50 amino acids in the G_C_ cytoplasmic tail truncated to allow the glycoproteins to traffic to the plasma membrane^45^ and RABV-G with the 333 attenuating mutation replacing VSV-G. CCHFV-M gene expressed by this vector does not contain GP38 in its virion because GP38 is cleaved from G_N_ and secreted from the cell^44,46^ thus, this vaccine is a negative control for the role of GP38-mediated protection. Lastly, VSV-ΔG-CCHFV-coM (GP38 + G_C_ + ) contains the same modified version of the M gene as GP38- G_C_+ but lacks its own VSV glycoprotein and incorporates GP38 into the virion due to a mutation in the cleavage site between GP38 and G_N_ as described previously^9^. Therefore, the GP38 + G_C_+ vaccine is a positive control for GP38-mediated protection.

All viruses were recovered, passaged twice and sequenced. The GP38+ G_C_+ virus developed two mutations, L517R and L518S, in the cleavage motif between GP38 and G_N_, as mentioned above, and the GP38+ G_C_- and GP38- G_C_+ viruses did not acquire any mutations.

### Incorporation of CCHFV Glycoproteins into Rhabdoviral vectors

To assess the expression of the CCHFV genes in the rhabdoviral vectors, we did immunofluorescence (IF) surface staining and flow cytometry analysis of Vero E6 cells infected with each virus. For IF, cells were infected at a multiplicity of infection (MOI) of 0.01. RABV infected cells were incubated for 72 h, and VSV infected cells were incubated for 24 h. For flow cytometry, Vero E6 cells were infected at MOI 10 for RABVs and MOI 5 for VSVs and incubated for 48 h or 8 h respectively. After infection, cells were fixed and stained with anti-RABV-G human monoclonal antibody 4C12 and either anti-CCHFV-G_C_ antibody 11E7 or anti-CCHFV-GP38 antibody 13G8. Surface staining of the infected cells showed that all the CCHFV proteins present in each of the rhabdoviruses were present on the cell surface (Fig. 2a–d, S1). RABV-G was detected from all the RABV-based vectors tested and the GP38- Gc+ virus which was engineered to contain RABV-G (Fig. 2a–d, S1).

To analyze the incorporation of the glycoproteins, we sucrose purified virions and separated the proteins on SDS Page protein gels. SYPRO™ Ruby staining showed incorporation of all the native rhabdoviral proteins in each virus (Fig. 2e, S2a, b). Western blotting for GP38 and G_C_ demonstrated that only GP38+ G_C_- and GP38+ G_C_+ viruses incorporate GP38, whereas GP38+ G_C_+ and GP38- G_C_+ viruses incorporate G_C_ (Fig. 2f, S2c). RABV-G was detected for the GP38- G_C_+ virus (Fig. 2f, S2c).

To analyze virus growth kinetics, we performed multi- and one-step growth curves for RABVs and one-step growth curves for VSVs. For multi-step growth curves, cells were infected at a low MOI of 0.01, and for one-step growth curves, cells were infected at a high MOI of 10. All CCHFV vaccine viruses showed slower growth kinetics compared to their parental vectors (Fig. 2g–i). Regardless of kinetics, all viruses grew to sufficient titers of at least 1 × 10^6^ focus forming units (ffu) for RABVs or plaque forming units (pfu) for VSVs.

These results show that rhabdoviruses with CCHFV glycoprotein genes are recoverable and incorporate the expected proteins into the virions.

### The Mucin-like domain is required for GP38 expression

We previously designed a vaccine that had GP38 with the RABV-G tail anchor but without the MLD, called BNSP333-GP38 (Fig. S3a). This virus was recovered, and characterization showed very poor expression of GP38. Immunofluorescence staining for GP38 on cells infected with BNSP333-GP85 showed very strong surface and intracellular expression of GP38, while cells infected with BNSP333-GP38 showed very minimal GP38 expression (Fig. S3b). Flow cytometry analysis of cells infected with BNSP333-GP38 or BNSP333-GP85 showed comparable levels of RABV-G expression between viruses, but only BNSP333-GP85 had high levels of GP38 (Fig. S3c). Finally, western blot for GP38 of sucrose purified virions showed that BNSP333-GP38 has virtually no incorporation of GP38 into virions compared to BNSP333-GP85 (Fig. S3d). These data show that the MLD is required for proper expression and incorporation of GP38 into rhabdoviruses.

### Immunogenicity of Rhabdoviral-based CCHFV vaccines

**Fig. 3:**
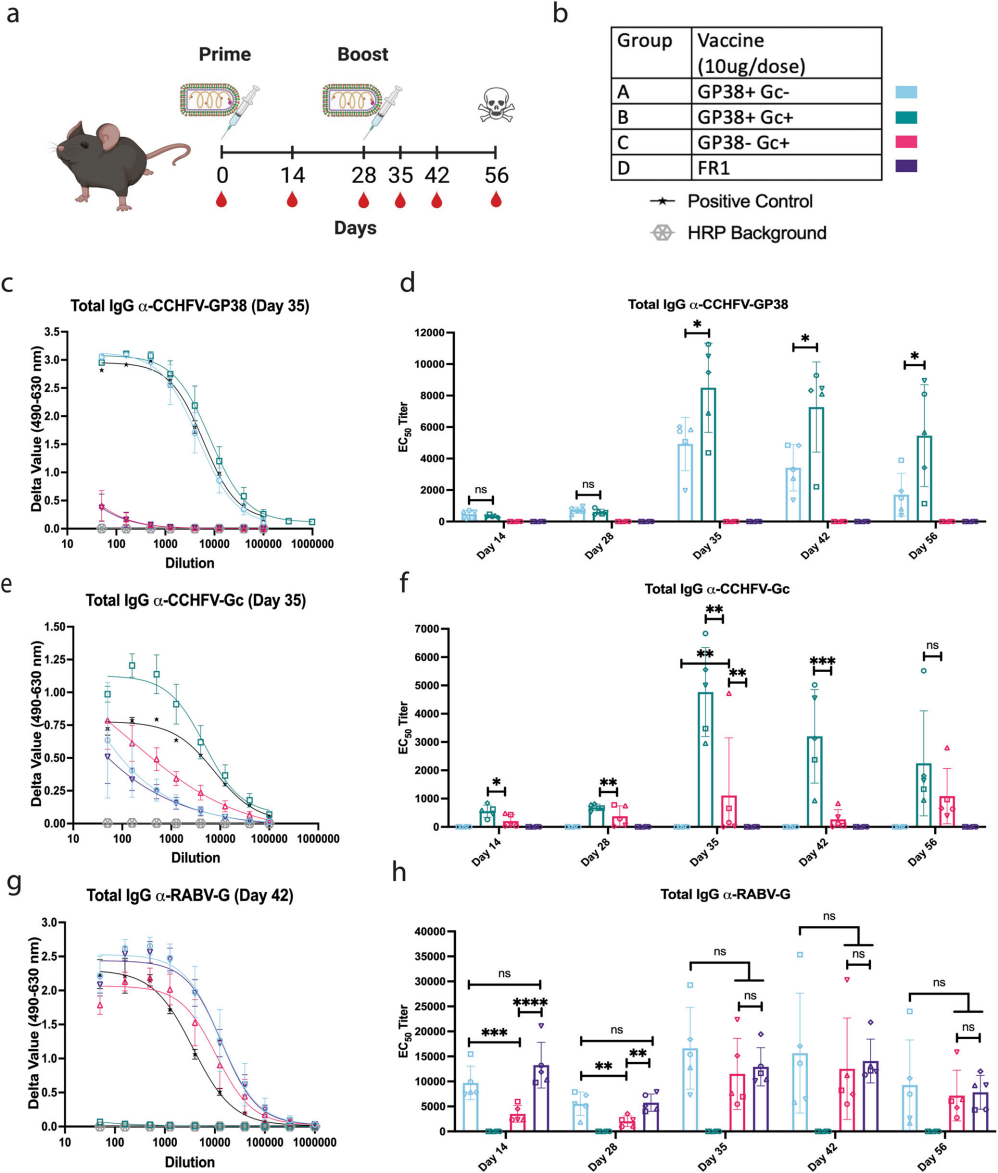
Rhabdoviral-based CCHFV vaccines elicit humoral responses against respective antigens. Immunogenicity study to look at antibody responses induced by each CCHFV vaccine. **a** Immunization and blood draw schedule for mouse studies. Groups of 5 mice were immunized with 10 µg/dose of BPL inactivated vaccines adjuvanted with 5 µg of PHAD in 2% SE per dose. Syringes represent immunizations, red blood drops indicate the days blood was taken and the skull denotes the conclusion of the study when the mice were sacrificed. Created with Biorender.com. **b** Table showing the vaccine groups used in this study and the symbols and colors used to denote each group and assay controls. **c**, **e**, **g** Group average ELISA curves for each antigen at the peak of the antibody response. Error bars represent standard deviation (SD). **d**, **f**, **h** EC_50_ ELISA titers over time for each antigen. Error bars indicate the mean with SD for groups of 5 mice with samples run in duplicate. An ordinary one-way ANOVA with Tukey’s Multiple Comparison Test was used to determine statistical differences between groups at each time point. All groups with detectable antibody titers have 4-star significance compared to groups where no antibody titers were detected (****P < 0.0001; ***P < 0.0002; **P < 0.0021; *P < 0.0332; ns not significant). **c**, **d** α-CCHFV-GP38 ELISAs, **e**, **f** α-CCHFV-G_C_ ELISAs, and **g**, **h** α-RABV-G ELISAs. ●, mouse 1; ■, mouse 2; ▲, mouse 3; ▼, mouse 4; ◆, mouse 5.

To investigate the immunogenicity of the vaccines, we immunized groups of 5 C57BL/6 (B6) mice with two doses, 28 days apart, of 10 µg of β-propiolactone inactivated vaccines (Fig. 3a, b). We used two groups per vaccine, one immunized with deactivated vaccine alone, the other containing deactivated vaccine adjuvanted with 5 µg of TLR-4 agonist synthetic Monophosphoryl Lipid A (MPLA), 3D(6 A)-PHAD (PHAD), in a 2% squalene-in-oil emulsion (SE). The mice were bled at various time points (Fig. 3a). All mice developed antibody responses against their respective antigens by day 14 post-immunization, which increased after the boost on day 28 and were maintained out to day 56 (Fig. 3). Using an adjuvant during vaccination typically improves the immune responses elicited by the vaccine^47–49^. Adjuvanted groups showed higher antibody responses for all vaccines against their respective antigens (Shown for GP38, Fig. S4). Thus, we decided to use adjuvants for all subsequent studies.

### Rhabdoviral-based CCHFV vaccines elicit a Th1-biased antibody response

**Fig. 4:**
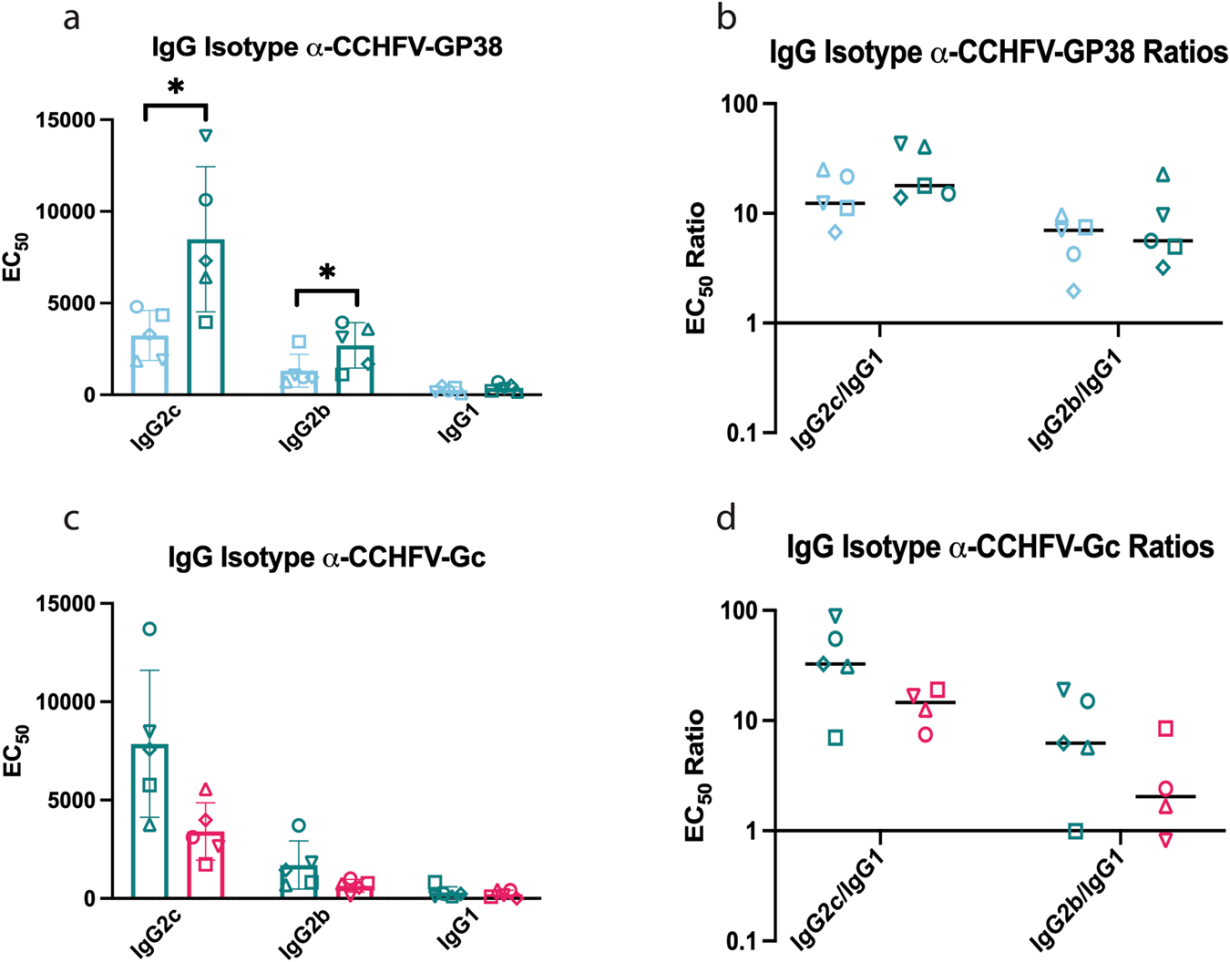
Rhabdoviral-based CCHFV vaccines induce a Th1-skewed humoral response. Isotype subclass ELISAs for each vaccine that had detectable antibodies in the CCHFV glycoprotein IgG Fc ELISAs. **a**, **c** EC_50_ antibody titers for each isotype subclass. Error bars indicate the mean with standard deviation (SD) for groups of 5 mice with samples run in duplicate. **b**, **d** Isotype ratios comparing EC_50_ titers of IgG2c or IgG2b to IgG1. Any animals with undetectable IgG1 were excluded from isotype ratio calculations. Lines represent median values. **a**, **b** GP38 isotype subclass ELISAs. **c**, **d** G_C_ isotype subclass ELISAs. Mann–Whitney test was used to determine statistical differences between groups for each isotype. (****P < 0.0001; ***P < 0.0002; **P < 0.0021; *P < 0.0332; ns not significant).

Th1 immune responses have been associated with strong anti-viral responses^50–53^. In B6 mice, IgG2b and IgG2c are associated with Th1 responses, while IgG1 is associated with Th2 responses^54^. We performed isotype subclass ELISAs using the day 56 sera from the immunogenicity study. All vaccines showed strong IgG2c and IgG2b antibody responses for their respective antigens, indicating a skew towards a Th1-associated response (Fig. 4).

### A VSV-based surrogate challenge model as a tool for determining CCHFV vaccine efficacy

CCHFV is a BSL-4 pathogen, which makes animal experiments with CCHFV expensive. Therefore, we developed a VSV-based surrogate challenge model for CCHFV using the GP38+ G_C_+ virus that replaces the native VSV-G with CCHFV-M. IFNAR^−/−^ mice are typically susceptible to both CCHFV and VSV^55,56^, so we first wanted to determine the ability of the surrogate challenge virus (GP38+ G_C_+ ) to cause disease in IFNAR^−/−^ mice. We challenged male mice intraperitoneally (I.P.) with either 5E5, 7.5E5 or 1E6 plaque forming units (pfu) of the GP38+ G_C_+ virus, and measured weight change and viral RNA copies in the blood via qPCR as indicators of disease. Pilot studies revealed that in IFNAR^−/−^ mice, this virus consistently causes high viremia and modest weight change regardless of challenge dose but is not uniformly lethal (Fig. S5). Thus, we decided to use a challenge dose of 5E5pfu and use viremia as the main indicator of disease in this surrogate challenge model.

**Fig. 5:**
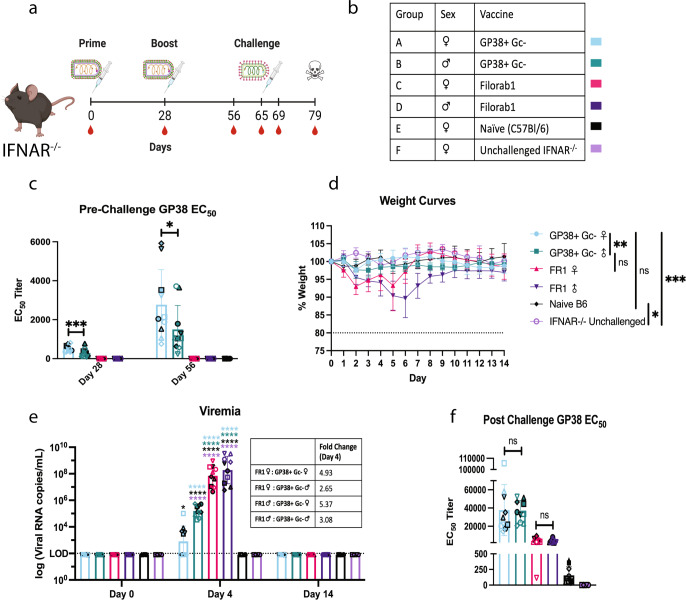
GP38 + G_C_- vaccine is protective in VSV-based surrogate challenge model. Challenge study to determine the utility of a VSV-based surrogate challenge virus when looking at vaccine protective efficacy. **a** Experimental timeline. Groups of 10 mice, 5 male and 5 female, were immunized with 10 µg/dose of BPL inactivated vaccines adjuvated with 5 µg of PHAD in 2% SE per dose as indicated by the syringe with the rhabdovirus containing multiple glycoproteins. Challenge of 5E5pfu of surrogate virus is indicated by the syringe with a VSV with a singular set of glycoproteins. Red blood drops indicate the days blood was taken, and the skull denotes the conclusion of the study when any surviving mice were sacrificed. Created with Biorender.com. **b** Table of vaccine groups and representative colors. GP38 EC_50_ titers pre-challenge (**c**) and post-challenge (**f**). Error bars indicate the mean with standard deviation (SD) for groups of 5 mice with samples run in triplicate. **d** Average group weight curves. Error bars indicate SD. **e** Viral RNA copies in the blood as determined by VSV-N qPCR. LOD, limit of detection. Error bars indicate the mean with SD. Results show the combination of two independent experiments; hollow symbols represent the first experiment and symbols with a black outline represent the second experiment. An ordinary one-way ANOVA with Tukey’s Multiple Comparison Test was used to determine statistical differences between groups at each time point for EC_50_ titers and viremia (**c**, **e**, **f**). Two-way ANOVA with Tukey’s Multiple Comparison Test was used to determine statistical differences between groups for the weight curves (**d**). All comparisons between groups not listed on the EC_50_ or weight change graphs had 4-star significant difference. (****P < 0.0001; ***P < 0.0002; **P < 0.0021; *P < 0.0332; ns not significant).

To test the utility of this challenge model for initial screening of vaccine efficacy, we immunized groups of male and female IFNAR^−/−^ mice with either GP38+ G_C_- vaccine or control FR1 vaccine, both adjuvanted with PHAD-SE (Fig. 5a, b). We included a naïve B6 group as a control for protection since these mice are not susceptible to this virus (Fig. 5b). All IFNAR^−/−^ mice immunized with the GP38 + G_C_- vaccine developed antibodies against CCHFV GP38, but we observed gender differences in antibody titer (Fig. 5c).

On day 65 post immunization, the vaccinated IFNAR^−/−^ and naïve B6 mice were challenged I.P. with 5E5pfu of the surrogate challenge virus (GP38 + G_C_ + ). Mice immunized with the GP38 + G_C_- vaccine showed minimal weight fluctuation post-challenge, while mice immunized with the FR1 vaccine showed modest weight loss (Fig. 5d, S6). One female and one male mouse from the FR1 immunized groups met endpoint euthanasia criteria on day 5 post-challenge. Mice vaccinated with the FR1 vaccine showed high viral RNA copies in the blood at 4 days post-infection, which were 3–5-fold higher compared to mice immunized with the GP38 + G_C_- vaccine, with some females completely clearing the virus (Fig. 5e). Mice vaccinated with the GP38 + G_C_- had a boost in GP38-specific antibody titers post-challenge (Fig. 5f).

These data show that the VSV-based surrogate challenge model for CCHFV can be used to test vaccine efficacy under BSL-2 conditions.

### Rhabdoviral-based CCHFV vaccine efficacy against wildtype CCHFV challenge

**Fig. 6:**
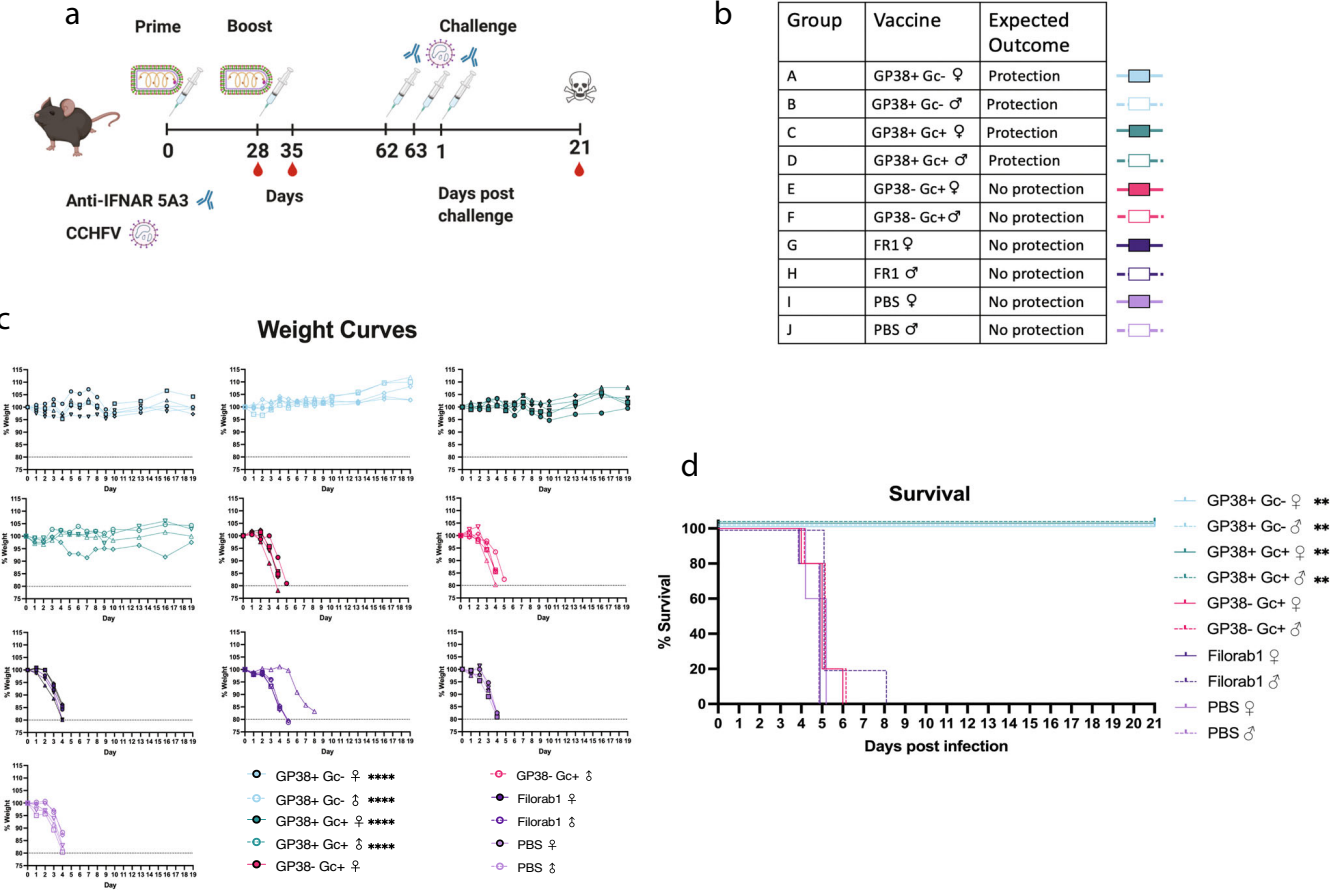
Vaccines that incorporate GP38 are protective against WT CCHFV Challenge. Challenge study to determine rhabdoviral-based CCHFV vaccine protective efficacy against CCHFV. **a** Experimental timeline. Groups of 10 mice, 5 male and 5 female, were immunized with 10 µg/dose of BPL inactivated vaccines adjuvanted with 5 µg of PHAD in 2% SE per dose as indicated by the syringe with the rhabdovirus. As denoted by the syringe with the antibody, mice were given mAb 5A3 24 h before and after challenge to make them susceptible to CCHFV. The syringe with the CCHFV indicates when mice were challenged with 1000pfu of strain IbAr10200 I.P. Red blood drops indicate the days blood was taken and the skull denotes the conclusion of the study when any surviving mice were sacrificed. Created with Biorender.com. **b** Table of vaccine groups, the expected outcome for that group and their representative colors. **c** Group average weight change over time. Error bars represent standard deviation. Dotted line indicates weight loss threshold for euthanasia. Statistics are two-way ANOVA compared to female PBS control group (****P < 0.0001). **d** Kaplan–Meyer survival curves. Log-rank Mantel-Cox test was used to determine the significance of survival of each group compared to the female PBS control group (**P < 0.0021).

To determine the protective efficacy of these rhabdoviral-based CCHFV vaccines, we performed a challenge experiment with wildtype (WT) CCHFV. B6 mice were immunized with 10 µg of vaccine/dose adjuvanted with PHAD-SE following the same prime/boost schedule used above for the immunogenicity studies (Fig. 6a). For this study, we utilized two groups of 5 mice per vaccine, one female and the other male, to detect any differences between the sexes. ELISAs against GP38 and G_C_ with sera collected at day 35 showed that all vaccines elicited strong antibody responses against the expressed CCHFV antigens, and there were no differences in antibody titers between sexes in the B6 mice (Fig. S7).

Given that WT mice are resistant to CCHFV infection, the immunized B6 mice were treated with anti-IFNAR monoclonal antibody mAb-5A3 to make them susceptible and then challenged I.P. with 1000pfu of CCHFV, strain IbAr10200. Mice vaccinated with either the GP38 + G_C_- or GP38 + G_C_+ vaccines maintained weight throughout the course of the challenge, while mice vaccinated with GP38- G_C_ + , FR1, or PBS showed dramatic weight loss starting by day 3 post challenge (Fig. 6c). All mice vaccinated with either GP38 + G_C_- or GP38+ Gc+ vaccines survived challenge out to day 21 and did not show any outward clinical signs of disease (Fig. 6d, S8). However, all mice vaccinated with either GP38- G_C_ + , FR1 or PBS succumbed to disease, with most mice reaching endpoint euthanasia criteria between days 4–6, except for one male mouse vaccinated with FR1 (Fig. 6d). There were no significant differences in weight loss or survival between mice of different sexes immunized with the same vaccine.

These results confirmed that only mice receiving vaccines containing GP38 (i.e., GP38 + G_C_- and GP38 + G_C_ + ) were protected against lethal CCHFV challenge.

### Vaccine-induced virus neutralization does not correlate with protection

**Fig. 7:**
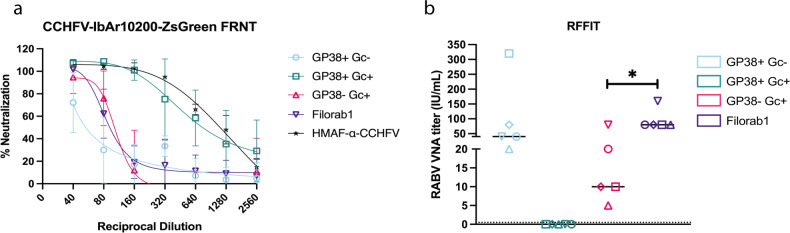
GP38 does not elicit CCHFV neutralizing antibodies. CCHFV and RABV neutralization assays. **a** Focus reduction neutralization test (FRNT) of a CCHFV strain IbAr10200 expressing ZsGreen (rCCHFV-ZsGreen) with sera from mice immunized with rhabdoviral vaccines. Hyperimmune mouse ascitic fluid (HMAF) against CCHFV served as a positive control. Error bars represent standard deviation (SD). **b** Rapid fluorescent focus inhibition test (RFFIT) with sera from mice immunized with rhabdoviral vaccines against RABV (strain CVS-11). Graph shows the RABV neutralizing IU/mL values for individual mice. Line represents the median values. Ordinary one-way ANOVA with Tukey’s Multiple Comparison Test was used to determine statistical differences between groups. All groups with detectable RABV neutralizing antibody titers have 4-star significance compared to groups where no antibody titers were detected (****P < 0.0001; ***P < 0.0002; **P < 0.0021; *P < 0.0332; ns not significant). Dotted line indicates 0.5IU/mL, the WHO suggested protective threshold. ●, mouse 1; ■, mouse 2; ▲, mouse 3; ▼, mouse 4; ◆, mouse 5.

To determine the CCHFV neutralizing capabilities of the rhabdoviral-based CCHFV vaccines, we performed a focus reduction neutralization test (FRNT) using a recombinant CCHFV expressing ZsGreen. Previous studies have suggested that protection from lethal challenge is achieved with neutralizing antibody titers of 1:160^9,12,13,32,33^, which in this assay, corresponds to 100% virus neutralization when using the hyper-immune mouse ascitic fluid (HMAF) control. The GP38 + G_C_+ vaccine had a FRNT_50_ of <1:1280 and showed neutralizing activity comparable to HMAF, with 100% virus neutralization at a 1:160 serum dilution (Fig. 7a). The GP38 + G_C_- and GP38- G_C_+ vaccines demonstrated minimal neutralization at a 1:160 serum dilution, similar to FR1 immunized control mice (Fig. 7a). These data indicate that vaccine-induced neutralizing antibodies are not the mechanism of protection for these vaccines.

We also analyzed a virus neutralization assay (VNA) for RABV. For RABV, induction of high levels of neutralizing antibodies post-vaccination correlates with protection^57^. As measured through the rapid fluorescent focus inhibition assay (RFFIT), mice immunized with GP38 + G_C_-, GP38- G_C_ + , or FR1 vaccines all showed high levels of RABV neutralizing antibodies, well above the 0.5 international units (IU)/mL threshold considered protective by the WHO (Fig. 7b). No RABV-neutralization was observed in mice immunized with the GP38 + G_C_+ vaccine (Fig. 7b).

### The GP38 + G_C_- vaccine does not induce GP38-specific T cell responses

The mechanism of protection for CCHFV has yet to be established. Therefore, we also analyzed the potential cellular responses to the GP38 + G_C_- vaccine. To this end, we collected splenocytes from mice vaccinated with either the GP38 + G_C_- or FR1 vaccine 2 weeks post-boost immunization and performed IFN-γ ELISpot assays. While we detected hundreds of IFN-γ spots produced by splenocytes stimulated with α-CD3 and α-CD28 antibodies, we saw low numbers (less than fifty) of IFN-γ spots produced by splenocytes stimulated with either a pool of GP38 peptides or GP38 protein (Fig. S9a). However, there was no significant difference between the number of IFN-γ spots produced by splenocytes from GP38 + G_C_- vaccinated mice and FR1 vaccinated mice (Fig. S9a).

To quantify the amount of IFN-γ produced by the splenocytes and to examine whether other cytokines are produced, supernatants from splenocytes from GP38 + G_C_- or and FR1 vaccinated mice were analyzed by a multiplex cytokine assay. As seen for the ELISpots, the splenocytes stimulated with α-CD3 and α-CD28 antibodies produced IFN-γ, however no IFN-γ was detected in supernatant from splenocytes stimulated with GP38 peptides (Fig. S9b). There were low levels of interleukin (IL)-1β, IL-2, IL-4, IL-6, monocyte chemoattractant protein (MCP)-1 and tumor necrosis factor (TNF)-α in splenocytes stimulated with GP38 peptides, however, there were no significant differences in cytokine levels between the GP38 + G_C_- and FR1 vaccinated mice (Fig. S9b). Therefore, we conclude that cellular immune responses against GP38 do not play a major role in protection against CCHFV for our vaccine.

## Discussion

CCHFV is an emerging disease for which no licensed treatments or vaccines are available. To this end, we developed an inactivated RABV-vectored CCHFV vaccine targeting the GP38 protein. This killed virus vaccine platform was safe to administer to both WT and immunocompromised (IFNAR^−/−^) mice and showed protection against lethal challenges in mice. Although GP38 is unique to the nairoviruses^58^, it has not been widely investigated as a potential vaccine target. However, it was recently shown that GP38 is indispensable for viral replication^31^ and GP38 targeted immune responses elicited protection against CCHFV challenge^15,37^. Thus, we decided to tailor our vaccine approach to target GP38.

We initially constructed a recombinant RABV containing a chimeric GP38/RABV G gene. This virus had poor expression and no GP38 incorporation, indicating that the MLD is required for GP38 processing. There is some evidence in the literature supporting this idea. Deleting the MLD changes GP38 localization and affects the incorporation of the structural glycoproteins into tc-VLPs^31^. However, we have shown with a live viral vector that the MLD is required for the proper processing of CCHFV GP38. We believe this is likely the reason that we observed better protection than the DNA vaccine targeting GP38 alone^15^. The GP38 DNA vaccine did not contain the MLD^15^ and thus GP38 was not sufficiently processed and unable to elicit the necessary immune responses for protection.

Moreover, we developed a BSL-2 surrogate challenge model to test CCHFV vaccine efficacy, given the challenges of performing such studies in BSL-4 labs. We previously demonstrated that such a model using a VSV with its native glycoproteins replaced with the LASV glycoproteins was useful for determining the mechanism of protection for a RABV-based LASV vaccine^23^. While the CCHFV model was not uniformly lethal in IFNAR^−/−^ mice, it did cause consistently high levels of viremia, an indicator of significant replication in the host. Additionally, we saw that the GP38 + G_C_- vaccine elicited protection in this surrogate challenge model, demonstrating its utility in analyzing vaccine protective efficacy. Of note, the results detected in the surrogate model translated well to the finding in the WT CCHFV challenge further indicating the model’s usefulness.

We hypothesized that only CCHFV-M targeting vaccines containing GP38 would be protective against CCHFV challenge. Our study confirmed the hypothesis that GP38 is required and sufficient for protection. We saw that both the GP38 + G_C_- and GP38 + G_C_+ vaccines protected 100% of mice against lethal CCHFV challenge, while our control mice, including the GP38- G_C_+ vaccinated mice, all succumbed to challenge. The full-length CCHFV-M gene or individual components have been tested as a CCHFV vaccine target in many vaccine strategies^8–10,15,32,33^. In line with our hypothesis that GP38 is required for protection when targeting CCHFV-M, vaccine strategies that use the entire M gene, such as DNA vaccines^15,16,32^ or live viral vectors^9,18^, have shown protection against WT CCHFV challenge. The study by Appelberg et al. (2021) indicated that their mRNA vaccine targeting the CCHFV glycoproteins only included G_C_ and G_N_, and this vaccine was shown to be protective^10^. However, the sequence of this vaccine encodes for the full CCHFV-M gene, meaning GP38 was included in this vaccine (personal correspondence D. Weissman, 2022). Conversely, those vaccine strategies that exclude GP38^13,33^ or do not develop immune responses against GP38^59^ are not protective against CCHFV challenge. Thus, we have confirmed that GP38 is an excellent vaccine target for CCHFV.

Our GP38 + G_C_- vaccine was protective against WT CCHFV challenge, with no visible weight loss or clinical signs. These results are comparable to other vaccine strategies targeting CCHFV-M that were protective against CCHFV challenge, including a live VSV-vectored vaccine^9^, live MVA-vectored vaccine^18^, CCHFV-M DNA vaccine^15^ and CCHFV-M mRNA vaccine^10^. However, our vaccine candidate has a few advantages over these other strategies. As mentioned above, our vaccine is a deactivated virus, making it safe to administer to various immunocompromised populations and pregnant women, unlike live virus vaccines. The DNA vaccine used a three dose immunization schedule^15^, while ours showed protection after only two doses. Major drawbacks of the mRNA platform are waning immunity^17^ and the necessity to store these vaccines at extremely cold temperatures. In contrast, the RABV vaccine has been shown to induce life-long immunity in humans^27^ and can be vaporized and remain stable at various temperatures, including storage at 50 °C for up to 2 weeks^60^. Additionally, the means of production for RABV-based vaccines already exists given that this vaccine has been produced and used for decades^61^.

Mice immunized with our various CCHFV vaccines showed strong antibody responses against their respective CCHFV glycoproteins and RABV-G with a skew towards a Th1 response. Two different CCHFV DNA vaccination strategies have investigated the types of antibody responses elicited from vaccination and showed that Th1 biased antibody responses were protective against CCHFV challenge^13,32^. Additionally, one of the studies demonstrated that vaccines eliciting a Th2 biased response were less protective compared to those eliciting a Th1 biased response^13^. The results of our vaccine study agree with these studies, further indicating that Th1 associated responses elicited by CCHFV vaccines are important for protection.

As far as we can tell, we are the only study to investigate whether there are sex differences for the immune responses elicited by CCHFV vaccines in mice. In B6 mice, there were no significant differences in the antibody responses elicited by these vaccines between males and females; however, there were significant differences in antibody titers between sexes in the IFNAR^−/−^ mice. It is well documented that there are differences in vaccine-elicited immune responses between males and females^62^. However, it is intriguing to see this difference in the IFNAR^−/−^ mice but not the WT mice, indicating the need to analyze a vaccine in different models. Regardless, our studies indicate this vaccine is effective in mice regardless of sex or immune status, something that is very important for an ideal vaccine candidate.

The correlates of protection for CCHFV are still unclear. For many viruses, including RABV^57^, neutralizing antibodies are considered the correlate of protection against viral infection. For CCHFV, this does not seem to be the case, as some vaccines eliciting high levels of neutralizing antibodies and treatment with certain neutralizing mAbs were not protective against CCHFV challenge^33,36,37^. The mechanism preventing certain neutralizing antibodies from protecting against CCHFV is unknown. One possibility is that the MLD is blocking epitopes on G_C_ that are targeted by neutralizing antibodies. This has been shown for the MLD of Ebola virus^63^ and the glycan shield on the Lassa virus glycoprotein^64^. Antibodies against GP38, which thus far have only been shown to have non-neutralizing functions, elicit protection against CCHFV challenge^36,37^. Additionally, there have been various vaccine strategies targeting the CCHFV nucleoprotein (NP) that are protective but do not induce production of neutralizing antibodies^10,65,66^. We also saw this in our study, with the GP38 + G_C_- vaccine eliciting an antibody response with minimal neutralizing activity but 100% protection of mice against WT CCHFV challenge. This provides further evidence that neutralizing antibodies are not a requirement for vaccine-mediated protection against CCHFV.

In contrast to other CCHFV-M vaccine platforms^10,15,16,66^, we did not observe significant levels of GP38-specific T cells in response to the rabies-based GP38 + G_C_- vaccine. It has been demonstrated that people receiving the RABV vaccine develop T cell responses to various RABV proteins^67–70^ and that these responses are mostly restricted to CD4^+^ T cells^67,71,72^. CD4^+^ T cell responses are much smaller in magnitude compared to CD8^+^ T cell responses^73–75^. While it seems unlikely from our data that the GP38+ G_C_- vaccine elicits GP38-specific CD8^+^ T cells, it is possible that it induces GP38-specific CD4^+^ T cells, but the response is too small to be measured in the standard IFN-γ ELISpot assay. The lack of CD8^+^ T cells is not unexpected for a deactivated vaccine as a live, replicating virus is normally required for induction of CD8^+^ T cell responses. Alternatively, the GP38+ G_C_- vaccine may not induce GP38-specific T cells at all, but elicits robust GP38-specific antibody responses because of T cell responses to the RABV proteins, similar to the mechanism of action for conjugate vaccines^76^.

Given the success of our GP38+ Gc- vaccine in this study, future testing is warranted to further characterize this vaccine and determine its utility in other preclinical models. Only two studies have investigated CCHFV vaccine mechanisms of protection: one, a live MVA-vectored CCHFV-M vaccine, required both humoral and cellular responses for protection^77^ the second a combination of RNA-based Venezuelan equine encephalitis virus replicon (repRNA) vaccines targeting CCHFV-S (repNP) and -M (repGPC), where the humoral response was determined to be the sole mechanism of protection for these vaccines^66^. Interestingly, the repGPC vaccine induced a weak antibody response was only 40% protective when administered alone^66^. Thus, it appears that the platform used plays a significant role in the type of immune response elicited against target antigens and their protective efficacy. A similar study investigating the protective effects of passive and/or adoptive transfer from mice immunized with our vaccine would be integral to understanding how our vaccine protects. Additionally, we saw that our GP38+ G_C_- vaccine had minimal CCHFV neutralization, thus it would be beneficial to investigate non-neutralizing antibody functions in combination with the passive transfer studies. Finally, testing the efficacy of our vaccine in the fully immunocompetent non-human primate (NHP) model^78^ is essential for confirming the vaccine’s efficacy and advancing this vaccine candidate to clinical trials.

In summary, this study shows that for CCHFV-M vaccines, GP38 is required and sufficient for protection. The GP38+ G_C_- (BNSP333-GP85) vaccine can progress to further testing in NHP and is an excellent candidate to be moved to the clinic.

## Methods

### Animals

C57BL/6 mice (Charles River) and B6.129S2-*Ifnar1*^*tm1Agt*^/Mmjax (The Jackson Laboratory) mice ages 6–10 weeks were used in this study. Both males and females were used. Mice used in this study were handled in adherence to the recommendations described in the *Guide for the Care and Use of Laboratory Animals* and the guidelines of the National Institutes of Health, the Office of Animal Welfare, and the United States Department of Agriculture. All animal protocols were approved by the Institutional Animal Care and Use Committee (IACUC) of Thomas Jefferson University (TJU) or University of Texas Medical Branch (UTMB) for experiments performed at each facility. The facilities where this research was conducted are fully accredited by the Association for Assessment and Accreditation of Laboratory Animal Care International. Mice were housed in cages, in groups of 5, under controlled conditions of humidity, temperature, and light (12 h light/12 h dark cycles). Food and water were available ad libitum. Animal procedures at TJU were conducted under 3% isoflurane/O_2_ gas anesthesia by trained personnel under supervision of veterinary staff.

### Cells

Vero (ATCC® E6™), 293 T (available from the Schnell laboratory), BSR (available from the Schnell laboratory) and BEAS-2B (ATCC® CRL-9609™) cells were cultured using DMEM (Corning®) with 5% fetal bovine serum (FBS) (Atlanta-Biologicals®) and 1% Penicillin-Streptomycin (P/S) (Gibco®). 293 F (ATCC® CRL-12585™) cells were cultured using FreeStyle™ 293 Expression Medium (Gibco®) with 2X Glutamax (Gibco®). Mouse neuroblastoma (NA) (available from the Schnell laboratory) cells were cultured using RPMI (Corning®) with 5% FBS and 1X P/S. Human hepatocarcinoma cells (HuH-7) (available from the Bente Laboratory) and SW-13 cells (available from the Bente Laboratory) were maintained in Dulbecco’s modified Eagle’s medium (DMEM) supplemented with 10% FBS (Invitrogen, Carlsbad, CA), 2mM L-glutamine (Invitrogen), and 1% P/S (Invitrogen), cumulatively called D10. All cells except 293 F were stored in incubators with 5% CO_2_ at 37 °C for normal cell culture or 34 °C for virus infected cells. 293 F cells were stored in incubators with 8% CO_2_ at 37 °C and shaking at 140 rpm.

### Viruses

RABV strain CVS-11 was produced in our laboratory on NA cells and is available upon request. A recombinant CCHFV, strain IbAr10200, ZsGreen reporter virus expressing the fluorescence tag on the N-terminus of the genomic S-segment ORF, designated rCCHFV-ZsGreen, was used for the fluorescence reduction neutralization test (kindly provided by Dr. Éric Bergeron of Centers for Disease Control and Prevention, Atlanta, GA). CCHFV strain IbAr10200 was obtained from the World Reference Collection of Emerging Viruses and Arboviruses at UTMB (WRCEVA, passaged 13 times in suckling mice and one time in Vero E6; Genbank sequences: NC005302, NC005300, and NC005301) and was passaged twice in SW-13 cells (ATCC, CCL-105) before use. All work with CCHFV was performed in a biosafety level 4 facility at the Galveston National Laboratory, University of Texas Medical Branch, Galveston, TX, in accordance with the approved Institutional Biosafety Committee protocols.

### Generation of Rhabdoviral vaccine vector cDNA

The human codon-optimized CCHFV-M, IbAr10200 strain^32^ (CCHFV-coM), used to develop the CCHFV vaccines was a generous gift from Dr. Aura Garrison (USAMRIID, Frederick, MD). All BNSP333^38^ and cVSV-XN^39^ vectors were kindly provided by Dr. Tiago Abreu-Mota (Thomas Jefferson University, Philadelphia, PA). The chimeric GP38 protein was cloned by first PCR amplifying the human Igκ signal sequence with primers GSP49 and GSP53 and GP38 with primers GSP54 and GSP55. This construct was cloned into a pDisplay vector with the addition of an HA tag through In-Fusion® cloning (Takara Bio). The GP38 gene containing the Igκ signal sequence was then PCR amplified with primers GSP68 and GSP69, and the RABV-G tail was amplified with primers GSP70 and GSP71. Through In-fusion®, these two PCR products were combined and cloned into a pCAGGS vector. This chimeric GP38 gene was then inserted into the BNSP333 vector using restriction sites BsiWI and NheI, and the plasmid was designated BNSP333-GP38. To produce the GP85 chimeric protein, the MLD gene was PCR amplified from the original CCHFV-coM gene using primers GSP84 and GSP85, and the GP38 chimeric gene was PCR amplified using primers GSP86 and GSP71, excluding the signal sequence. This chimeric GP85 gene was cloned into a pCAGGS vector with In-fusion® cloning and finally cloned into the BNSP333 vector using restriction sites BsiWI and NheI. This resulting plasmid was designated BNSP333-GP85 (GP38+ G_C_-). All CCHFV-coM genes were PCR amplified to have 50 amino acids in the G_C_ cytoplasmic tail truncated as previously described^45^. Primers GSP03 and GSP20 (GP38+ G_C_+ ) or GSP21 (GP38- G_C_+ ) were used to PCR amplify the CCHFV-coM for the VSV vectors, and GSP06 and GSP07 were used to PCR amplify RABV-G containing the R333E mutation (RVG-333) for the VSV vector. CCHFV-coM was inserted into the VSV vectors using either MluI and NotI (GP38- G_C_+ ) or MluI and NheI (GP38+ Gc + ) restriction sites. RVG-333 was inserted into the VSV vector containing CCHFV-coM using NotI and NheI restriction sites. The resulting plasmids were designated VSV-ΔG-CCHFV-coM-RVG (GP38- G_C_+ ) and VSV-ΔG-CCHFV-coM (GP38+ G_C_+ ). The sequences of these three plasmids were confirmed by sequencing using primers GSP08, GSP09, RP591, RP592, RP1325, and RP1327 for the RABV vector and GSP08-GSP19, VPF5, and VP9R for the VSV vectors. Primer sequences are listed in Table 1.

**Table 1:** Primer Sequences.

Primer	Direction	Sequence	Use
GSP03	Forward	5'-CGATCTGTTTACGCGTGCCACCATGCACATCAGCC-3'	PCR amplification of CCHFV-coM for insertion into VSV vectors. Primer contains MluI restriction site.
GSP06	Forward	5'-AGATATCACGCTCGAGGCCACCATGGTTCCTCAGG-3'	PCR amplification of RVG-333 for insertion into VSV vector. Primer contains NotI restriction site.
GSP07	Reverse	5'-GAAGAATCTGGCTAGCTTACAGTCTGGTCTCACCCCC-3'	PCR amplification of RVG-333 for insertion into VSV vector. Primer contains NheI restriction site.
GSP08	Reverse	5'-CTCGCCGGTGATGAAGAACT-3'	CCHFV-coM sequencing primer.
GSP09	Forward	5'- ACCCTGTGAGAAACCTGCTG-3'	CCHFV-coM sequencing primer.
GSP10	Reverse	5'- TTGATCACGCAGTCGGTGAA-3'	CCHFV-coM sequencing primer.
GSP11	Forward	5'- CCTGAAGGCCAGCATCTTCA-3'	CCHFV-coM sequencing primer.
GSP12	Reverse	5'- GCAGTAGGGGCAGATGTTGT-3'	CCHFV-coM sequencing primer.
GSP13	Forward	5'- GGCGATATCCTGGTGGACTG-3'	CCHFV-coM sequencing primer.
GSP14	Reverse	5- CAGTGTCTGCAGTAAGGGC-3'	CCHFV-coM sequencing primer.
GSP15	Forward	5'-TGCCCTTACTGCAGACACTG-3'	CCHFV-coM sequencing primer.
GSP16	Reverse	5'- ATGTTTCTGGGCTCGGACAG-3'	CCHFV-coM sequencing primer.
GSP17	Forward	5'- TCAACGTGCAGTCCACCTAC-3'	CCHFV-coM sequencing primer.
GSP18	Reverse	5'- TCCTCCTCGCTACAGCTCTT-3'	CCHFV-coM sequencing primer.
GSP19	Forward	5'- AAGAGCTGTAGCGAGGAGGA-3'	CCHFV-coM sequencing primer.
GSP20	Reverse	5'-GCTAGCTTAGCCTCTGGTTCTCCG-3'	PCR amplification of CCHFV-coM for insertion into VSV-ΔG vector for surrogate challenge virus. Primer contains NheI restriction site.
GSP21	Reverse	5'-GCGGCCGCTTAGCCTCTGGTTCTCCG-3'	PCR amplification of CCHFV-coM for insertion into VcoM vector for vaccine. Primer contains NotI restriction site.
GSP42	Reverse	5'-CATAGTCATCTTCATTGA-3'	Sequencing primer for RVG in VSV-ΔG-coM-RVG.
GSP49	Forward	5'-GCCGCCAGTGTGCTGGAATTCGCCACCATGGAGACAGACACA-3'	PCR amplification of signal sequence for GP38 chimeric gene.
GSP53	Reverse	5'-tcttcaggttGTCACCAGTGGAACCTGGAACC-3'	PCR amplification of signal sequence for GP38.
GSP54	Forward	5'-CACTGGTGACaacctgaagatggagatca-3'	PCR amplification of GP38 for GP38 with a signal sequence.
GSP55	Reverse	5'-AACATCGTATGGATAGTCGACGGACCCGGTGCTGGCCTT-3'	PCR amplification of GP38 for GP38 with a signal sequence.
GSP66	Forward	5'-TAATACGACTCACTATAGGGGGACAGCCTGATGACATTG-3'	Primer for IVT of VSV-N.
GSP67	Reverse	5'-TCTGGTGCATACAAACCT-3'	Primer for IVT of VSV-N.
GSP68	Forward	5'-CAAAGAATTCCGGAACGTACGGCCACCATGGAGACAGACACA-3'	PCR amplification to put the GP38 with the signal sequence into a pCAGGS plasmid.
GSP69	Reverse	5'-CCGAGGATTCGGACCCGGTGCTGGCCTT-3'	PCR amplification to put the GP38 with the signal sequence into a pCAGGS plasmid.
GSP70	Forward	5'-CACCGGGTCCGAATCCTCGGTTATCCCCC-3'	PCR amplification of RABV-G 51 amino acids of the ectodomain (ED51), transmembrane domain (TM) and cytoplasmic tail (CT) to make the chimeric GP38.
GSP71	Reverse	5'-GAGGGAAAAAGATCTGCTAGCTTACAGTCTGGTCTCACCCCC-3'	PCR amplification of RABV-G 51 amino acids of the ectodomain (ED51), transmembrane domain (TM) and cytoplasmic tail (CT) to make the chimeric GP38.
GSP72	Forward	5'-CCTCTGCCGACTTGGCACAA-3'	Primer for qPCR of VSV-N.
GSP73	Probe	5'-CCGGAGGATTGACGACTAATGCACCGCCACAAGGCAG-'	Primer-probe for qPCR of VSV-N.
GSP74	Reverse	5'-CCGAGCCATTCGACCACATC-3'	Primer for qPCR of VSV-N.
GSP84	Forward	5'-CAAAGAATTCCGGAACGTACGATGCACATCAGCCTGATGTACGC-3'	PCR amplification of MLD to create the chimeric GP85.
GSP85	Reverse	5'-TCTTCAGGTTCCGCTTGCTCCTGTTGGTGG3'	PCR amplification of MLD to create the chimeric GP85.
GSP86	Forward	5'-GAGCAAGCGGAACCTGAAGATGGAGATCATCCTGA-3'	PCR amplification of chimeric GP38 to create the chimeric GP85.
GSP87	Forward	5'-AATTCCGGAACGTACGGCCACCATGGAGCTGAGG-3'	PCR amplification of human furin gene to insert into pCAGGS plasmid.
GSP88	Reverse	5'- AAAAAGATCTGCTAGCTTAGAGGGCGCTCTGGTC-3'	PCR amplification of human furin gene to insert into pCAGGS plasmid.
RP591	Forward	5'-GGAGGTCGACTAAAGAGATCTCACATAC-3'	Sequencing of foreign gene in BNSP333 vector.
RP592	Reverse	5'-TTCTTCAGCCATCTCAAGATCGGCCAGAC-3'	Sequencing of foreign gene in BNSP333 vector.
RP1325	Forward	5'-GTTATGGTGCCATTAAACCGCTG-3'	Sequencing of RVG in BNSP333 vector.
RP1327	Reverse	5'-TCTCCAGGATCGAGCATCTT-3'	Sequencing of RVG in BNSP333 vector.
VP5F	Forward	5'-GCGTGGGTCCTGGATTCTAT-3'	Sequencing of foreign gene in VSV vector
VP9R	Reverse	5'-ATCGAGGGAATCGGAAGAGAAT-3'	Sequencing of foreign gene in VSV vector

### Recovery of recombinant viruses

Recombinant RABV and VSV vaccines were recovered using established cDNA reverse genetics^79,80^. X-tremeGENE 9 (MilliporeSigma®) in Opti-MEM (Gibco®) was used to co-transfect the respective full-length viral cDNA along with pTIT plasmids encoding RABV-N, -P, and -L or VSV-N, -P, and -L proteins, with the addition of RABV-G for the VSV surrogate challenge virus and pCAGGs plasmids expressing T7 RNA polymerase in 293 T cells in poly-l-lysine coated 6-well plates. The supernatants of RABV transfected cells were harvested every 3 days, and VSV transfected cell supernatants were harvested every 2 days. Presence of infectious RABV was detected by immunostaining for RABV-N with 1:200 dilution of fluorescein isothiocyanate (FITC) anti-RABV-N monoclonal globulin (Fujirebio®, product #800–092) or for virus-induced cytopathic effect (CPE) in the case of VSV.

### Viral production and titering

GP38+ G_C_-, GP38- G_C_+ , GP38+ G_C_+ , Filorab1^81^ (generous gift of Dr. Drishya Kurup, Thomas Jefferson University, Philadelphia, PA), BNSP333^38^, VSV-GFP (plasmid provided by Dr. Tiago Abreu-Mota), VSV-ΔG-RABV-G and SPBN^38^ viruses were grown and titered on Vero cells. Specifically, Vero cells were cultured with VP-SFM (Gibco®) supplemented with 1% P/S, 2X GlutaMAX™ (Gibco®) and 10 mM HEPES buffer (Corning®) and infected with a multiplicity of infection (MOI) of 0.01 for Filorab1, BNSP333, and VSV-GFP and 0.001 for GP38+ G_C_-, GP38- G_C_+ , and GP38+ G_C_+ . GP38+ G_C_+ to be used in the surrogate challenge model was grown on BSR cells in DMEM supplemented with 5% FBS and 1% P/S, infected at MOI 0.001. VSV-ΔG-RABV-G and SPBN were grown on BEAS-2B cells in OptiPRO™ SFM (Gibco™), supplemented with 1% P/S, 2X GlutaMAX™ (Gibco®) and 10 mM HEPES buffer (Corning®), and infected with a multiplicity of infection (MOI) of 0.01. Viruses were harvested every 3 days with VP-SFM media replacement until viral titers started to decrease for RABVs or until 80% cytopathic effect was detected for VSVs. RABV titering was performed by limiting dilution focus-forming assay using a 1:200 dilution of FITC anti-RABV-N monoclonal globulin (Fujirebio®; catalogue number: 800–092)^82^. VSV titers were determined by standard plaque forming assay, where virus was serially diluted, then 2% methyl cellulose was overlaid onto the wells and plates were left to incubate for 1–3 days until plaques became visible^83^.

### Purification and virus inactivation

To produce inactivated GP38+ G_C_-, GP38- G_C_+ , GP38+ G_C_+ , and Filorab1 vaccines, viral supernatant was concentrated, sucrose purified^84^, and inactivated^81^. Viral supernatants with the highest titers were pooled for each virus and concentrated at least 5x in an Amicon® 300 mL stirred cell concentrator using a 500 kDa exclusion PES membrane (MilliporeSigma®). Concentrated supernatants were then overlaid onto a 20% sucrose cushion and centrifuged at 76,755 x *g* for 2 h. Virions pellets were resuspended in TEN buffer (100 mM Tris base, 50 mM NaCl, 2 mM EDTA in ddH_2_O) with 2% sucrose and incubated overnight (O.N.) at 4 °C. β-propiolactone (BPL) (MilliporeSigma®) was added at a 1:2000 dilution for inactivation. Samples were left at 4 °C O.N. shaking and then incubated the following day at 37 °C for 30 min to hydrolyze the BPL. Virus inactivation was confirmed by inoculated supernatant with 10 µg of inactivated virions was passaged in T25 flasks of Vero cells; cells were fixed and stained with a 1:200 dilution of FITC anti-RABV-N (Fujirebio®, product #800–092) or monitored for cytopathic effect^25^.

### Immunofluorescence

3E5 Vero cells were seeded on glass coverslips in a 12-well plate and infected the next day at an MOI of 0.01 with the respective viruses. After 72 h (RABV viruses) or 24 h (VSV viruses), cells were washed in 1X DPBS and fixed for 10 mins in 2% paraformaldehyde (PFA) in 1X DPBS for surface staining. Those slides to be used for intracellular staining were then fixed for an additional 15 mins in 2% PFA with 0.1% Triton™ X-100 (Sigma-Aldrich®). Subsequently, cells were washed 2–3 times with 1X DPBS and blocked in 1X DPBS with 5% FBS for 1 h at room temperature or overnight at 4 °C. Cells were then probed for 1 h at room temperature with primary antibodies in 1X DPBS with 1% FBS, specifically, anti-RABV-G 4C12 (provided by Scott Dessain, Lankenau Institute for Medical Research, Wynnewood, PA) at 4 µg/mL, with either anti-G_C_ 11E7 (BEI resources, NR-40277) at 3.2 µg/mL or anti-GP38 13G8 (BEI resources, NR-40294) at 2.4 µg/mL. Cells were washed once with 1X DPBS and incubated with 2.5 µg/mL of anti-mouse AF568 (ThermoFisher, A-11004) and 2.5 µg/mL of anti-human AF647 (ThermoFisher, A48279) in 1X DPBS with 1% FBS for 45 mins at room temperature. Cells were then washed 5 times with 1X DPBS, mounted onto slides using mounting media containing 4',6-diamidino-2-phenylindole (DAPI) (ProLong™ Glass Antifade Mountant, Invitrogen™ catalog number: P36980), and stored O.N. at room temperature in the dark. Slides were visualized the next day using a Nikon Ti-E microscope with Nikon A1R Laser Scanning confocal camera with the Plan Fluor 40x/1.3 objective lens on the NIS-Elements C software for multi-dimensional experiment acquisition and analysis at 23 °C. Color channels were processed (channels separated for individual images and merged for merged images) using ImageJ software (OSS NIH).

### Glycoprotein FACS analysis

A total of 8E5 Vero cells for RABVs or 3E5 Vero cells for VSVs were seeded in 6-well plates. The following day, cells were infected with RABVs at MOI 10 for 48 h or left uninfected (control). Two days later, cells were infected with VSVs at MOI 5 for 8 h. Medium was then aspirated, and cells were washed once with 1X DPBS. Cellstripper® (Corning™, catalog number 25–056-Cl) was added to each well for 5–10 min to remove the cells from the well. Cells were then transferred to 15 mL conical tubes and centrifuged at 400 x *g* for 5 min. Cells were resuspended in 100 µL per 8E5 cells of 2% PFA in 1X PBS, seeded in a 96-well round bottom plate with 8E5 cells per well, and fixed for 10 min. Cells were centrifuged at 250 x *g* for 3 min and washed three times in 200 µL FACS buffer (10% FBS and 0.05% NaN_3_) per well. Cells were stained in 100 µL of primary antibody mixture containing anti-RABV-G 4C12 (provided by Scott Dessain, Lankenau Institute for Medical Research, Wynnewood, PA) at 4 µg/mL and either anti-G_C_ 11E7 (BEI resources, NR-40277) at 3.2 µg/mL or anti-GP38 13G8 (BEI resources, NR-40294) at 2.4 µg/mL in FACS buffer O.N. at 4 °C. The next day, cells were washed twice with 200 µL FACS buffer and then stained with 100 µL of secondary antibody mixture containing goat anti-mouse BV510 (BioLegend®, 405331) at 0.2 µg/100 µL and goat anti-human AF647 (ThermoFisher, A48279) at 2.5 µg/mL in FACS buffer for 2 h at room temperature. Cells were then washed three times in 200 µL FACS buffer and transferred to FACS tubes in a total of 400 µL FACS buffer. Cells were analyzed for GFP emission to detect GFP expression (i.e., VSV-GFP infection) in the FITC channel, BV510 emission to detect CCHFV-G_C_ or GP38 in the BV510 channel, and AF647 emission to detect RABV-G in the allophycocyanin (APC) channel using a BD FACSCelesta™ Cell Analyzer. Data analysis was performed using FlowJo software (Treestar, Ashland, OR).

### SDS PAGE protein gel and western blot

Sucrose purified virus particles and purified CCHFV glycoproteins were denatured with Urea Sample Buffer (125 mM Tris-HCl [pH 6.8], 8 M urea, 4% sodium dodecyl sulfate, 0.02% bromophenol blue) and reduced with 2-mercaptoethanol (CAS No. 60–24–2, Millipore Sigma®) and boiling at 95 °C for 10 min. However, samples to be probed with any of the anti-CCHFV antibodies were left unreduced, as these antibodies are conformational. 1 µg of samples for total protein analysis were resolved on a 10% SDS-PAGE gel and stained O.N. with SYPRO™ Ruby Protein Gel Stain (ThermoFisher Scientific). 1 µg of samples for western blot analysis were resolved on a 10% SDS-PAGE gel and transferred onto a nitrocellulose membrane in Towbin buffer (192 mM glycine, 25 mM Tris, 20% methanol). Blots were then blocked in 5% milk dissolved in PBS-T (0.05% Tween® 20 [MilliporeSigma®]) at room temperature for 1 hr. Next, membranes were incubated with primary antibody O.N. at 4 °C. Antibodies were made in a solution of 5% bovine serum albumin (BSA) in PBS. Anti-G_C_ 11E7 (BEI resources, NR-40277) was used at a dilution of 320 ng/mL, anti-GP38 13G8 (BEI resources, NR-40294) was used at a dilution of 240 ng/mL, and anti-RABV-G 4C12 (provided by Scott Dessain, Lankenau Institute for Medical Research, Wynnewood, PA) was used at 2 µg/mL dilution. The next day the blots were washed with PBS-T and incubated with horseradish peroxidase (HRP)-conjugated anti-mouse (Jackson ImmunoResearch, 115–035–146) or human IgG (SouthernBiotech, 2040–05) at 1:40,000 dilution in PBS-T for blots probed with 11E7, 1:20,000 dilution in PBS-T for blots probed with 13G8 or 1:20,000 in PBS-T for blots probed with 4C12. Proteins were detected with SuperSignal West Dura Chemiluminescent substrate (Pierce®) and imaged on the FluorChem R system (proteinsimple®).

### Multi-step and one-step growth curves

Vero E6 cells were seeded in 6-well plates at 7E5 cells/well. The following day, cells were checked for 70% confluency and then infected in serum free medium at MOI 0.01 for multi-step growth curves or MOI 10 for one-step growth curves. After 2 h of incubation, the media was aspirated, and the infected cells were washed 2X with 1X DPBS (Corning®). DMEM supplemented with 5% FBS and 1% P/S was added to each well, and the first sample of 200 µL was taken from each well. Samples were taken every 24 h until 120 h post-infection for RABVs and at 2, 4, 6, 8, 12, 24, 36, and 48 h post-infection for VSVs. Each viral sample was titered in triplicate as described above in the *Viral production and titering* section.

### Immunizations

Groups of five 6- to 10-week-old male and female C57BL/6 mice were immunized intramuscularly (I.M.) with 10 µg BPL-inactivated virus (see Fig. 3a for dose schedule) formulated alone in PBS or with the addition of Synthetic Monophosphoryl Lipid A (MPLA), 3D(6 A)-PHAD, in a squalene-in-oil emulsion (PHAD-SE), at a dose of 5 µg PHAD and 2% SE. Each immunization was administered as a total of 100 µL, with 50 µL injected in each hind leg muscle. Serum was collected through retro-orbital bleeds performed under isoflurane anesthesia on days 0, 14, 28, 35, and 42, with the final bleed on day 56.

### Production of ELISA antigens

RABV-G antigen was produced by stripping the glycoprotein off of virions^53^. BEAS-2B cells were infected with VSV-ΔG-GFP-RABV-G (for RABV vaccines) or SPBN (for VSV vaccines) in Opti-PRO (Gibco®). Viral supernatants were concentrated and purified as described above in the purification section. After sucrose purification, viral pellets were resuspended in TEN buffer (100 mM NaCl, 100 mM Tris, 10 mM EDTA pH7.6) containing 2% OGP (Octyl-β-D-glucopyranoside) detergent and incubated for 30 min at room temperature while shaking. This mixture was centrifuged at 3000 x *g* for 10 min, supernatant collected and further centrifuged at 45,000 x *g* for 90 min. Supernatant was collected and analyzed for presence of antigen via western blot with anti-RABV-G antibody.

CCHFV-G_C_ HA-tagged antigen was prepared through transfection^25^. Subconfluent T175 flasks of 293 T cells that were poly-l-lysine coated were transfected with a eukaryotic expression vector (pDisplay) encoding for each individual CCHFV glycoprotein with the cleavage sites and transmembrane regions removed, specifically amino acids 1040 to 1631 of CCHFV-M, fused to a C-terminal hemagglutinin (HA) peptide. Supernatant was collected 1 week after transfection, clarified by centrifugation, and filtered through a 0.45 μm filter before being loaded onto an equilibrated anti-HA agarose column (Pierce) containing either a 2.5 mL or 5 mL agarose bed volume. The supernatant was allowed to bind to the column overnight at 4 °C. The following day, the column was washed with 10-bed volumes of PBS-T, and bound HA-tagged glycoprotein was eluted with 5–10 mL of 0.4 mg/mL HA peptide in PBS. Fractions were collected and analyzed for the presence of G_C_ glycoprotein through western blot with CCHFV-G_C_ 11E7 antibody. Peak fractions were pooled and dialyzed against PBS in 10,000 molecular weight cutoff dialysis cassettes (MWCO) (Thermo Scientific™) to remove excess HA peptide. After dialysis, the protein was quantified by nanodrop 2000c spectrophotometer and/or bicinchoninic acid (BCA) assay. Halt ™ Protease Inhibitor Cocktail (Thermo Scientific™, catalog number: 78430) was added for a final concentration of 1X and sodium azide (NaN_3_) added for a final concentration of 0.05% before freezing the protein in small aliquots at −80 °C.

CCHFV-GP38 Strep-tagged antigen was prepared from an enhanced expression vector (pEEV) containing the sequence for CCHFV-GP85 strain IbAr10200 from amino acids 22 to 515, with a N-terminal FLAG and His tag and a C-terminal Strep-Tag II (referred to as pEEV-HisFlag-GP85–10200-Strep) (generously provided by Dr. Éric Bergeron at the Centers for Disease Control, Atlanta, GA). The plasmid pLEX307-FURIN-puro (ID # 158460), containing the human furin gene was ordered from AddGene. This gene was then PCR amplified with primers GSP87 and GSP88 and cloned into a pCAGGS vector through In-Fusion™ cloning. 293 F cells were grown in FreeStyle™ 293 Expression Medium (Gibco®) with 2X Glutamax (Gibco®) and seeded at 3×10^6^ cells/mL in Erlenmeyer flasks. The next day, cells were transfected using FectoPRO® (Polyplus transfection™) transfection reagent following the reagent manual with slightly altered conditions. The pEEV-HisFlag-GP85–10200-Strep and pLEX307-FURIN-puro plasmids were transfected at a ratio of 4:1 in a total of 0.8 µg plasmid DNA for each ml of culture. This co-transfection with the furin plasmid was to ensure that the MLD was cleaved from GP38. Media for the transfection complexes was 1/10 of the total culture volume and 1.5 µL of FectoPro reagent was used per µg of DNA. 4 h after transfection, FectoPRO® booster was added in an equivalent amount to that of DNA (i.e., 0.8 µg/mL DNA = 0.8 µL FectoPRO® booster/mL). Cells were incubated until cell viability sharply declined, typically around 3 days post transfection. The supernatant was then harvested, spun down for 30 mins at 4000 x *g* and filtered through a 0.45 μM filter before being loaded onto a column with a 2 mL bed volume of Strep-Tactin®XT resin (IBA Lifesciences). The supernatant was allowed to bind to the column overnight at 4 °C. The following day, the column was washed with 5 column bed volumes of 1X Buffer W (IBA Lifesciences) and then eluted with 6×0.5 column bed volumes of 1X Buffer BXT (IBA Lifesciences), collected as 0.5 mL fractions. Fractions were analyzed for the presence CCHFV-GP38 through western blot with CCHFV-GP38 13G8 antibody as described in the Western blot section above. The protein was quantified by nanodrop 2000c spectrophotometer and/or bicinchoninic acid (BCA) assay. Halt ™ Protease Inhibitor Cocktail (Thermo Scientific™, catalog number: 78430) was added for a final concentration of 1X and sodium azide (NaN_3_) added for a final concentration of 0.05% before freezing the protein in small aliquots at -80 °C.

### Enzyme-linked immunosorbent assay (ELISA)

Individual mouse serum was analyzed by ELISA for the presence of IgG specific to CCHFV-GP38, -G_C_, and RABV-G. Antigens were diluted in coating buffer (15 mM Na_2_CO_3_, 35 mM NaHCO_3_ [pH 9.6]) at a concentration of 100 ng/well (1 ng/µL) for GP38, 150 ng/well (1.5 ng/µL) for G_C_, and 50 ng/well (0.5 ng/µL) for RABV-G, and then 100 µL was added to each well of 96-well immulon 4HBX plates (Nunc®) and incubated O.N. at 4 °C. The following day, plates were washed three times with PBS-T (0.05% Tween 20 in 1X PBS), blocked for 2 h (5% milk in PBS-T), and washed again three times with PBS-T. Sera or control mAbs were diluted in three-fold serial dilutions (starting with a 1:50 dilution or higher dilutions of 1:150, 1:450, or 1:1350 for sera that did not reach endpoint titer) down the plate in 1X PBS with 0.5% BSA and incubated O.N. at 4 °C. Plates were then washed three times with PBS-T and 100 µL secondary antibody HRP conjugated goat anti-mouse IgG Fc (SouthernBiotech 1033–05) at a concentration of 50 ng/mL for GP38 and G_C_, and 25 ng/mL for RABV-G in PBS-T was added to each well and incubated for 2 h at room temperature. For isotype subclass ELISAs, the appropriate secondary antibody (Jackson ImmunoResearch, IgG1–115–035–205; IgG2b—115–035–207; IgG2c—115–035–208) was used at the same concentration as the IgG Fc-specific secondary antibody. Then plates were washed three times with PBS-T, and 200 µL of o-phenylenediamine dihydrochloride (OPD) substrate (ThermoFisher®) was added and left incubating for 15 min for GP38 and G_C_ and 13 min for RABV-G. The reaction was stopped by adding 50 µL of 3 M Sulfuric acid (H_2_SO_4_). Optical density was determined at 490 nm (OD490) and 630 nm (OD630) and delta values calculated subtracting the background OD630 readings from the OD490 readings. ELISA data was analyzed with GraphPad Prism 9 using a sigmoidal nonlinear fit (4PL regression curve) model to determine the half maximal Effective Concentration (EC_50_) serum or antibody titer. An accurate EC_50_ value cannot be calculated without a full curve, therefore samples without a proper curve are considered to have no detectable antibodies against that antigen and have a reported EC_50_ of 1. Isotype ratios were calculated by taking either the IgG2c or IgG2b EC_50_ value, dividing it by the IgG1 EC_50_ value. For those samples where there was no detectable IgG1 antibodies, no isotype ratio could be calculated. Positive controls (when available) for each assay were as follows: α-CCHFV-GP38 13G8 at a starting concentration of 1 µg/mL for IgG Fc and IgG2b GP38 ELISAs; α-CCHFV-GP38 10E11 (BEI Resources, NR-40276) at a starting concentration of 1 µg/mL for IgG1 GP38 ELISAs; α-CCHFV-G_C_ 11E7 at a starting concentration of 2 µg/mL for IgG Fc Gc ELISAs; α-RABV-G 1C5 (Abcam, Ab82460) at a starting concentration of 1 µg/mL for IgG Fc RABV-G ELISAs.

### Surrogate CCHFV challenge virus pathogenicity

Groups of five 8–10-week-old male interferon α/β receptor 1 knockout (IFNAR^−/−^) mice were infected with either 5E5, 7.5E5 or 1E6 pfu of GP38+ G_C_+ virus I.P. (200 µL total) to determine the parameters needed for use as a challenge model. The virus was diluted in PBS for all doses. Mice were weighed daily and monitored for signs of disease until day 14 post-infection. Mice that lost more than 20% of their starting weight or appeared moribund were humanely euthanized. Blood was collected at days 0, 4, and 14 to be used for in a VSV-N qPCR to look for viremia.

### Surrogate CCHFV challenge model in mice

Groups of five 8- to 10-week-old male and female IFNAR^−/−^ mice were immunized I.M. with 10 µg of BPL inactivated GP38+ G_C_- or FR1 vaccines adjuvanted with 5 µg PHAD in 2% SE at days 0 and 28 (Fig. 7a). On day 65, mice were injected with 5E5pfu of GP38+ G_C_+ diluted in PBS as determined above. Mice were sacrificed: (1) when weight loss reached ≥ 20% or (2) if severe clinical signs of disease were observed. Terminal bleeding was collected upon sacrifice when possible. Mice were bled at days 0, 4, and 14 to look for viremia in a VSV-N qPCR.

### RNA extraction

50 µL of whole blood was added to 300 µL of TRIzol LS Reagent (Life Technologies) and 50 µL of DPEC water, or 250 µL of virus supernatant was added to 750 µL of TRIzol LS Reagent. The protocol for RNA extraction of biological fluids with TRIzol LS Reagent was used up to the phase separation step. Then the protocol from the PureLink RNA Mini Kit (Ambion) was used for the remainder of the extraction. A NanoDrop (Fisher) was used to measure the concentration and quality (260/280 ratios) of extracted RNA.

### Measuring surrogate challenge virus viremia via quantitative Real-Time polymerase chain reaction (qPCR)

First, VSV-N RNA was prepared to act as a standard for the qPCR. RNA was isolated from GP38+ G_C_+ virus and cDNA produced using the One-Step RT PCR (SuperScript IV, Thermo Fisher Scientific) with primers GSP66 and GSP67. This cDNA was used to produce RNA standards via in-vitro transcription using the MEGAscript® T7 Kit (Invitrogen™) followed by the MEGAclear™ Transcription Clean-Up Kit (Invitrogen™). The qPCR was then run following the protocol for TaqMan Fast Virus 1 Step Master Mix reagent (ThermoFisher), using 5 µL of RNA per reaction, primers GSP72 and GSP74, and probe GSP73 with a 60 °C annealing temperature. Any day 0 samples showing detectable viral RNA were considered contaminated and not reported. Full primer and probe sequences are listed in Table 1.

### Wildtype CCHFV challenge in B6 mice with IFNAR blockade

Mice were challenged with 1000pfu of CCHFV strain IbAr10200 by intraperitoneal (I.P.) route^85^. Virus was diluted in a total volume of 0.1 ml of PBS (Gibco). All mice were injected I.P. with a total of 2.5 mg of anti-IFNAR 1 (mAb-5A3; Leinco Technologies Inc.) diluted in PBS 24 h before (2.0 mg) and 24 h after infection (0.5 mg) in a total volume of 0.2 ml. Mice were observed at least daily and weighed for the first 10 days daily and then every 3 days.

### Wildtype CCHFV FRNT

Mouse sera were serially diluted 1:2 in serum-free DMEM then incubated with rCCHFV-ZsGreen virus for 1 h on ice. The mixture was inoculated onto wells of HuH-7 cells and incubated for 1 h at 37 °C with 5% CO2. Cells were then supplemented with D10 and incubated until 48 h post infection. Relative fluorescence of each well was measured on a GFP plate reader. Wells inoculated with rCCHFV-ZsGreen virus only served as the control for maximum fluorescence, and wells inoculated with serum-free DMEM without virus served as the control for background fluorescence. Percent virus neutralization was calculated from the percent of fluorescence reduction from serum plus virus wells compared to virus only wells. IC_50_ values were determined using a four parameter, variable slope, nonlinear regression model in GraphPad PRISM.

### Rapid Fluorescent Focus Inhibition Test (RFFIT)

RFFIT neutralization assay was performed to look for RABV neutralizing antibodies^86^. Serum was heat inactivated at 56 °C for 30 mins. NA cells were seeded at 3E4 cells per well in a 96-well plate. Two days later, serum samples were diluted in a 2-fold dilution series in Opti-MEM in 96-well plates at a starting dilution of 1:40 (unless stated otherwise). The US standard rabies immune globulin (WHO Standard) was used at a starting dilution of 2IU/mL. A dilution of CVS-11 previously determined to produce 90% infection was added to each well with either sera or the WHO Standard and incubated for 1 h at 34 °C. The media in the plates with the NA cells was then replaced by the sera/virus mixture and incubated for 2 h at 34 °C. This media was aspirated, and fresh Opti-MEM was added. Plates were incubated for 22 h at 34 °C and then fixed with 80% acetone and stained with a 1:200 dilution of FITC-conjugated anti-RABV-N antibody for at least 4 h. The Reed-Muench method was used to calculate 50% endpoint titers, which were subsequently converted to international units (IU) per milliliter through comparison to the WHO standard.

### T cell ELISpot

Mice were immunized following the same prime/boost schedule described above and spleens were harvested for T cell ELISpot 2 weeks after the boost immunization. Spleens were processed and incubated for 20 h with a GP38 peptide pool, GP38 whole antigen and/or ebolavirus glycoprotein (EBOV-GP) antigen^53^, all at a final concentration of 2.5 μg/mL. As a positive control, splenocytes were incubated with a mixture of anti-CD3 (Clone 17A2, BD Biosciences, 555273) at 1 μg/mL and anti-CD28 (Clone 37.51, Biolegend, 102102) at 5 μg/mL. The GP38 peptide pool contained 65 peptides, 15-mers with an 11 amino acid overlap (JPT Peptide Technologies), spanning across the entire GP38 protein. Mouse IFN-γ ELISpot kits (Mabtech) were used to develop the plates following the manufacturer’s instructions. Plates were then read on an AID *v*Spot Spectrum plate reader using AID EliSpot software version 7. The number of spots was normalized to the amounts for 10^6^ cells and background corrected by subtracting the number of spots seen in the unstimulated wells for each sample. Any sample that had a negative value after background correction was reported as 0 spots.

### Cytokine multiplex assay

For the cytokine multiplex assay, samples were prepared identical to the T cell ELISpot, except in a regular 96-well flat-bottom plate. After 20 h of incubation, plates were centrifuged, and supernatant was collected. The supernatant was shipped to Eve Technologies Corporation (Calgary, Alberta) for analysis. Specifically, the multiplexing analysis was performed using the Luminex™ 200 system (Luminex, Austin, TX, USA). Ten markers were simultaneously measured in the samples using Eve Technologies’ Mouse Focused 10-Plex Discovery Assay® (MilliporeSigma, Burlington, Massachusetts, USA) according to the manufacturer’s protocol. The 10-plex consisted of Granulocyte-macrophage colony-stimulating factor (GM-CSF), interferon (IFN)-γ, interleukin (IL)-1β, IL-2, IL-4, IL-6, IL-10, IL-12p70, monocyte chemoattractant protein (MCP)-1, and tumor necrosis factor (TNF)-α. Assay sensitivities of these markers range from 0.4–10.9 pg/mL for the 10-plex. Individual analyte sensitivity values are available in the MilliporeSigma MILLIPLEX® MAP protocol. Like the ELISpot data, the cytokine amounts were normalized to the amount for 10^6^ cells and background corrected by subtracting the cytokine amounts from the unstimulated wells for each sample. Any sample with a negative concentration of cytokine after background correction was reported as 0 pg/mL. Samples under the limit of detection were reported as not detected (ND) for an entire group, or individual symbols were not included on the graph.

### Statistical analysis

All statistical analysis was performed using GraphPad Prism 9 on log transformed data. For growth curves, each time point was compared to the parental vector control using the ordinary one-way ANOVA with the Tukey Multiple Comparison Test. The Mann Whitney test was used for comparison within two groups at each timepoint for all ELISA EC_50_ data and IU/mL RFFIT data. For groups analysis at each time point of ELISA EC_50_ titers, IU/mL RFFIT data, and qPCR viral RNA copies, an ordinary one-way ANOVA was used with a post-Hoc analysis using Tukey Multiple Comparison Test with a 95% confidence interval. To look at the differences in group average weight change over time for the surrogate challenge virus, a two-way ANOVA was used with Tukey’s Multiple Comparisons Test. A two-way ANOVA was used with a Dunnett multiple comparisons test to compare differences in weight loss over time to the control female PBS group for the WT CCHFV challenge. The log-rank Mantel-Cox test was performed to compare differences in survival to the control female PBS group. All mouse studies were performed with groups of five mice unless otherwise stated. While groups of 5 are sufficient for statistical analysis, two groups for each vaccine (one male and one female) were tested in each challenge study, further strengthening the results. Additionally, the WT CCHFV challenge model is 100% lethal and power analysis showed that a 20% difference in survival can be detected with a p value of 0.05. To compare differences between the vaccine groups for both the IFN-γ ELISpot and cytokine multiplex analysis, multiple Mann–Whitney tests were performed.

### Materials availability

Upon request, further information, resources, and reagents are available from the authors pending an executed MTA as well as biosafety approval of the requesting institutions(s).

### Reporting summary

Further information on research design is available in the Nature Research Reporting Summary linked to this article.

## Supplementary information

Supplemental Material

REPORTING SUMMARY

## Data Availability

All data are available upon request to the lead contact author. No proprietary software was used in the data analysis.
