# Supplementary material for: GP38 as a vaccine target for Crimean-Congo hemorrhagic fever virus
Source: NPJ Vaccines. 2023 May 20;8:73. doi: 10.1038/s41541-023-00663-5 (PMC10199669; doi:10.1038/s41541-023-00663-5)
Supplement: Supplementary file 1 — Supplemental Material [file 41541_2023_663_MOESM1_ESM.pdf]

Supplemental Material

a

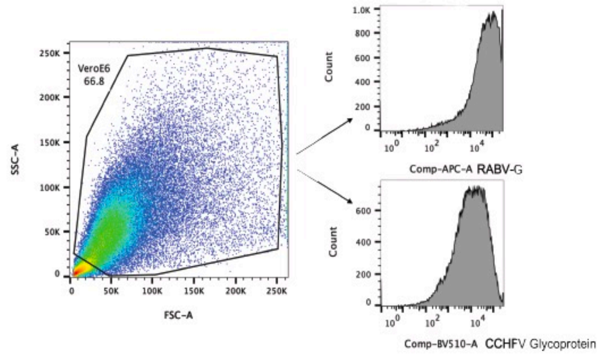

b

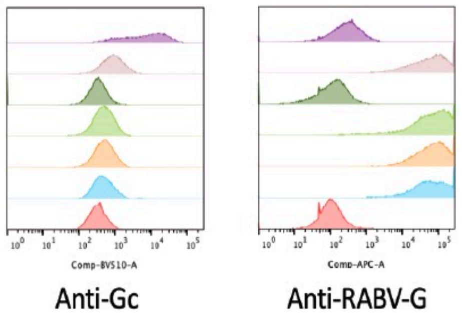

	Sample	Geometric Mean α-Gc	Geometric Mean α-RABV-G
	GP38+ Gc+	5607	241
	GP38- Gc+	807	41379
	VSV-GFP	253	93.6
	GP38+ Gc-	450	43554
	FR1	476	54374
	BNSP333	467	50060
	Mock	312	95.5

c

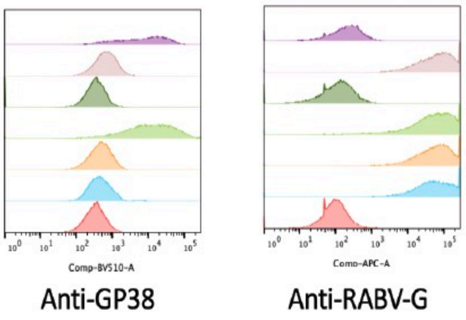

	Sample	Geometric Mean α-GP38	Geometric Mean α-RABV-G
	GP38+ Gc+	5494	163
	GP38- Gc+	610	46231
	VSV-GFP	259	96.1
	GP38+ Gc-	8456	34397
	FR1	433	42987
	BNSP333	442	42845
	Mock	313	86

Supplemental Figure 1. Gating Strategy and raw data for figure 2C and 2D. (A) Gating strategy for quantifying antigen expression on the surface of infected cells. (B&C) Histograms and numerical values of flow cytometry staining of infected cells. Vero E6 cells were infected with RABVs at MOI 10 for 48hrs or VSVs at MOI 5 for 8hrs and then fixed. Cells were then probed with α-RABV-G 4C12 and α-CCHFV-Gc 11E7 (B) or α-CCHFV-GP38 13G8 (C) and analyzed by flow cytometry. Experiment was performed multiple times, and this is one representative experiment.

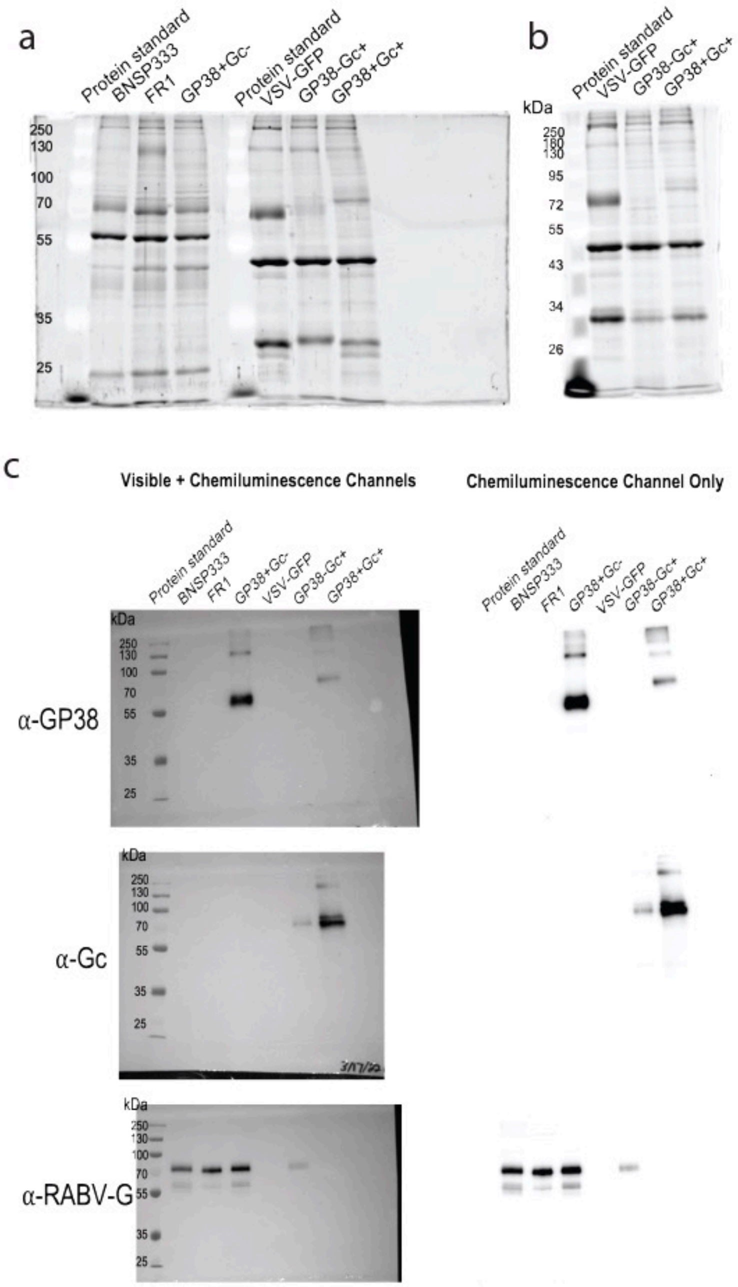

Supplemental Figure 2. Raw files for figure 2E and 2F*.* (A, B) SDS PAGE protein gel of sucrose purified virions. 1µg of sucrose purified virions were run on the gel and stained with SYPRO™ Ruby stain. (A) Gel that was used for RABVs in figure 2E. (B) Gel that was used for VSVs in figure 2E. (C) Western blot of sucrose purified virions. 1µg of sucrose purified virions were run on an SDS PAGE gel and transferred to a nitrocellulose membrane for western blotting. Blots were either probed with α-CCHFV-GP38 13G8 (top panel), α-CCHFV-Gc 11E7 (middle panel) or α-RABV-G 4C12 (bottom panel). Image on the left is the merge of both visible and chemiluminescent channels to be able to see the ladder. Image on the right is just the chemiluminescent channel.

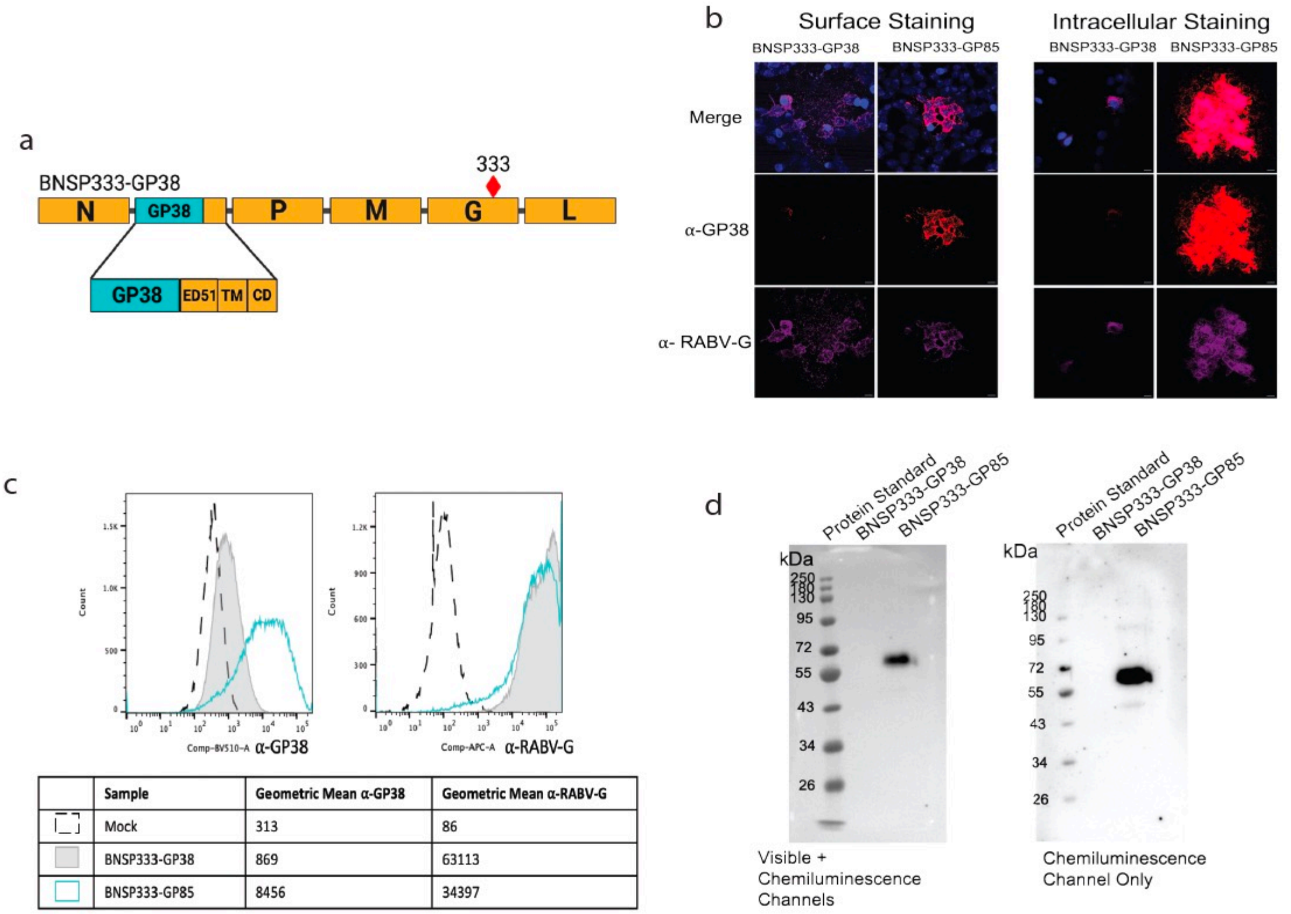

	Sample	Geometric Mean α-GP38	Geometric Mean α-RABV-G
	Mock	313	86
	BNSP333-GP38	869	63113
	BNSP333-GP85	8456	34397

Supplemental Figure 3. The Mucin-Like Domain is important for GP38 Processing. (A) Schematic of BNSP333-GP38 vaccine construct with chimeric GP38/RABV-G pop out to show the individual domains of the RABV-G tail. Created with Biorender.com. (B) Immunofluorescence staining of infected cells. Vero E6 cells were infected with either BNSP333-GP38 or BNSP333-GP85 at MOI 0.01 for 72hrs and then fixed. Cells used for Intracellular staining were permeabilized with 0.1% Triton™ X-100 following fixation. Cells were then stained with α-RABV-G 4C12 (purple) and α-CCHFV-GP38 13G8 (red) and mounted with mounting media containing a nuclear DAPI stain (blue). In the merged images, areas where there is overlapping expression of RABV-G and CCHFV-GP38 are pink. Images were taken at 40X magnification with a 2X zoom. Scale bars represent 10μm. (C) Histograms and numerical values of flow cytometry staining of infected cells. Vero E6 cells were infected with either BNSP333-GP38 or BNSP333-GP85 at MOI 10 for 48hrs and then fixed. Cells were then probed with α-RABV-G 4C12 and α-CCHFV-GP38 13G8 and analyzed by flow cytometry. Experiment was performed multiple times, and this is one representative experiment. (D) Western blot of sucrose purified virions. 1μg of sucrose purified virions were run on an SDS PAGE gel and transferred to a nitrocellulose membrane for western blotting. Blots were probed with α-CCHFV-GP38 13G8. The image on the left is the merge of the visible and chemiluminescent channels to show the visible ladder markers, while the image on the right is just the chemiluminescent channel alone.

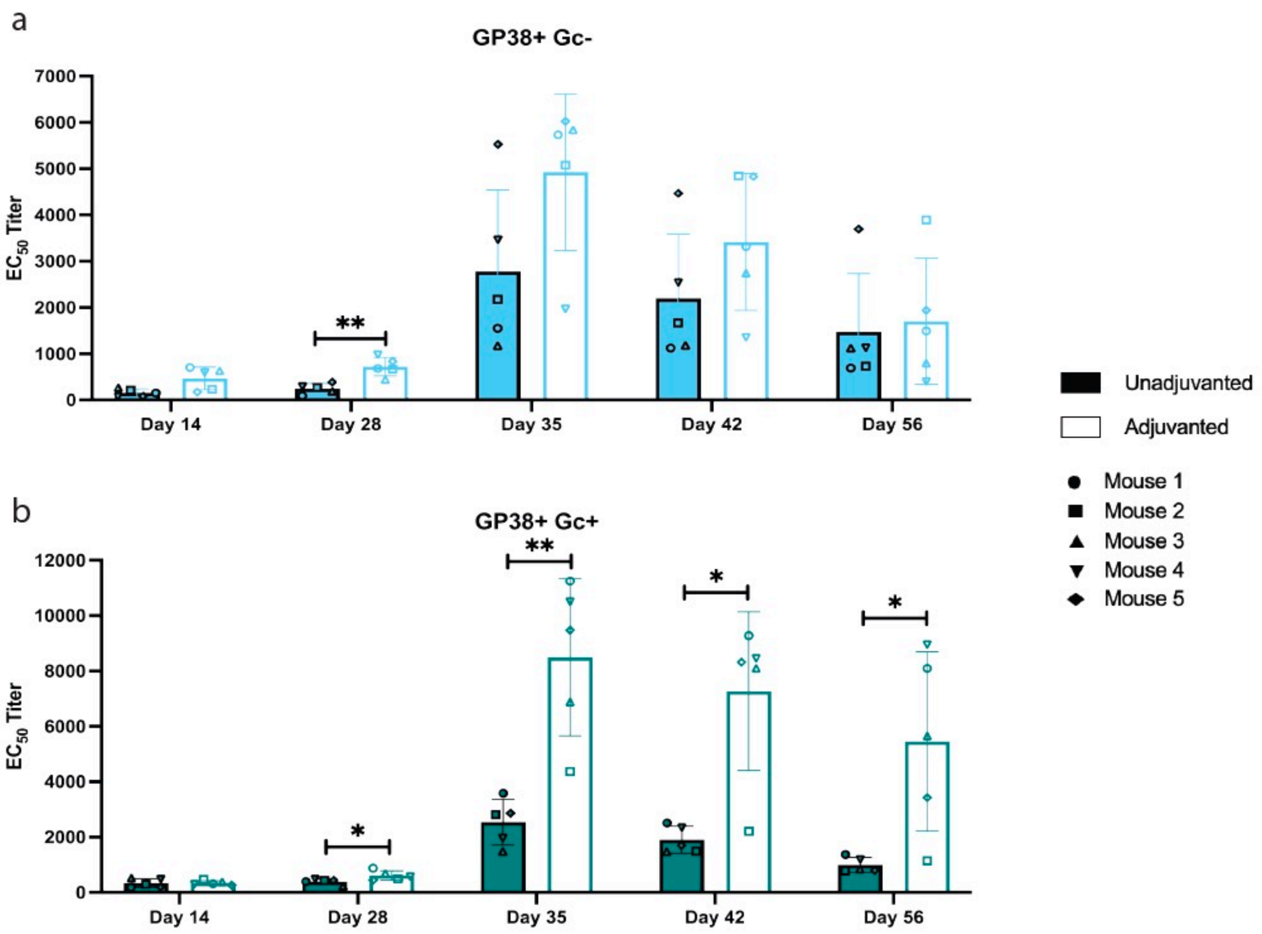

Supplemental Figure 4. The adjuvant PHAD-SE boosts the antibody response to the rhabdboviral vaccines. α-CCHFV-GP38 total IgG ELISAs for sera from GP38+ Gc- (A) and GP38+ Gc+ (B) immunized mice. Groups of 5 female mice were immunized with 10µg per dose of BPL inactivated vaccine either with or without PHAD-SE adjuvant. EC_{50} titers are compared over time between mice receiving unadjuvanted (solid symbols) and adjuvanted (clear symbols) vaccines. Error bars indicate the mean with standard deviation (SD) for groups of 5 mice with samples run in duplicate. The Mann-Whitney nonparametric t Test was used to determine statistical differences between groups at each time point. (*****P* < 0.0001; ****P* < 0.0002; ***P* < 0.0021; **P* < 0.0332; ns = not significant).

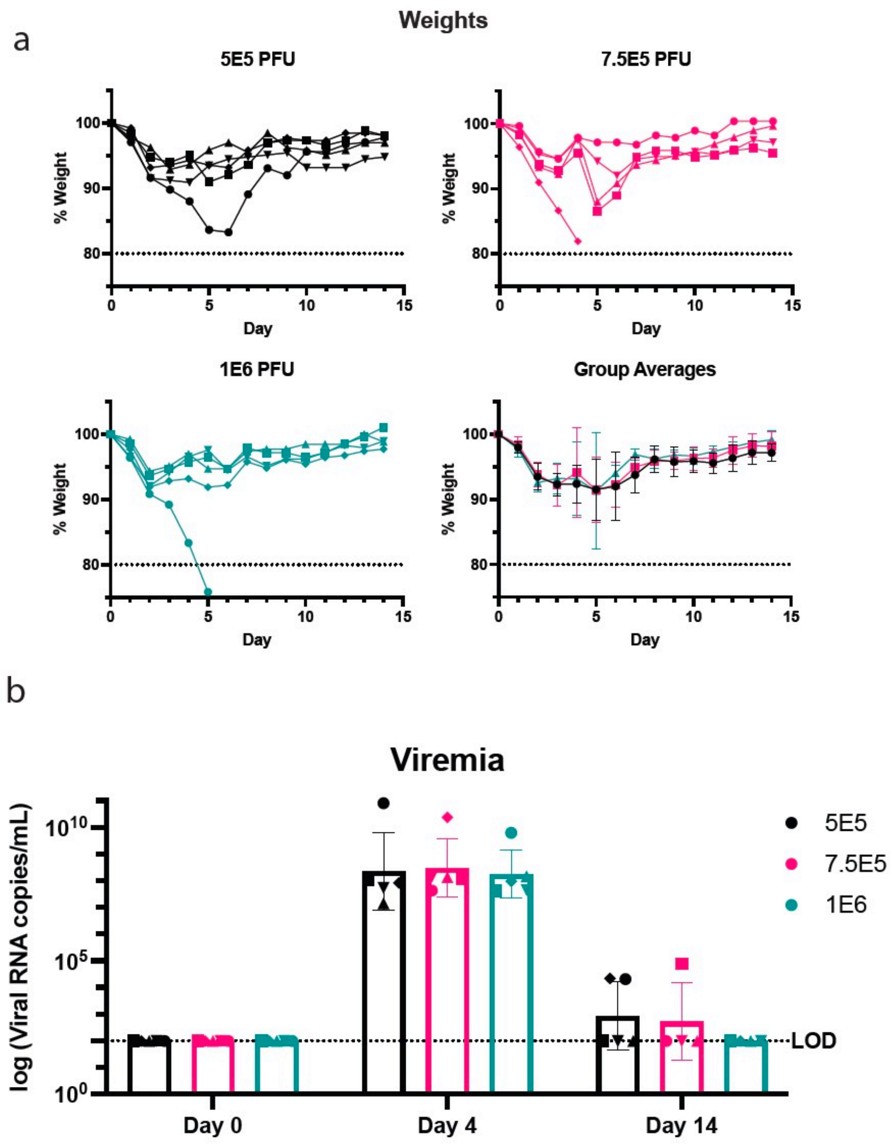

Supplemental Figure 5. Pilot study of the surrogate challenge virus in IFNAR$^{-/-}$ mice. Groups of 5 male IFNAR$^{-/-}$ mice were challenged I.P. with either 5e5, 7.5e5 or 1e6 pfu of the surrogate challenge virus (GP38+ Gc+). (A) Weight curves that represent the percent change in weight from the day of challenge. Dotted line represents 20% weight loss, the point at which mice were euthanized. Error bars indicate SD. (B) Levels RNA copies in the blood of mice as determined by qPCR for VSV-N. LOD, limit of detection. Error bars indicate the mean with SD.

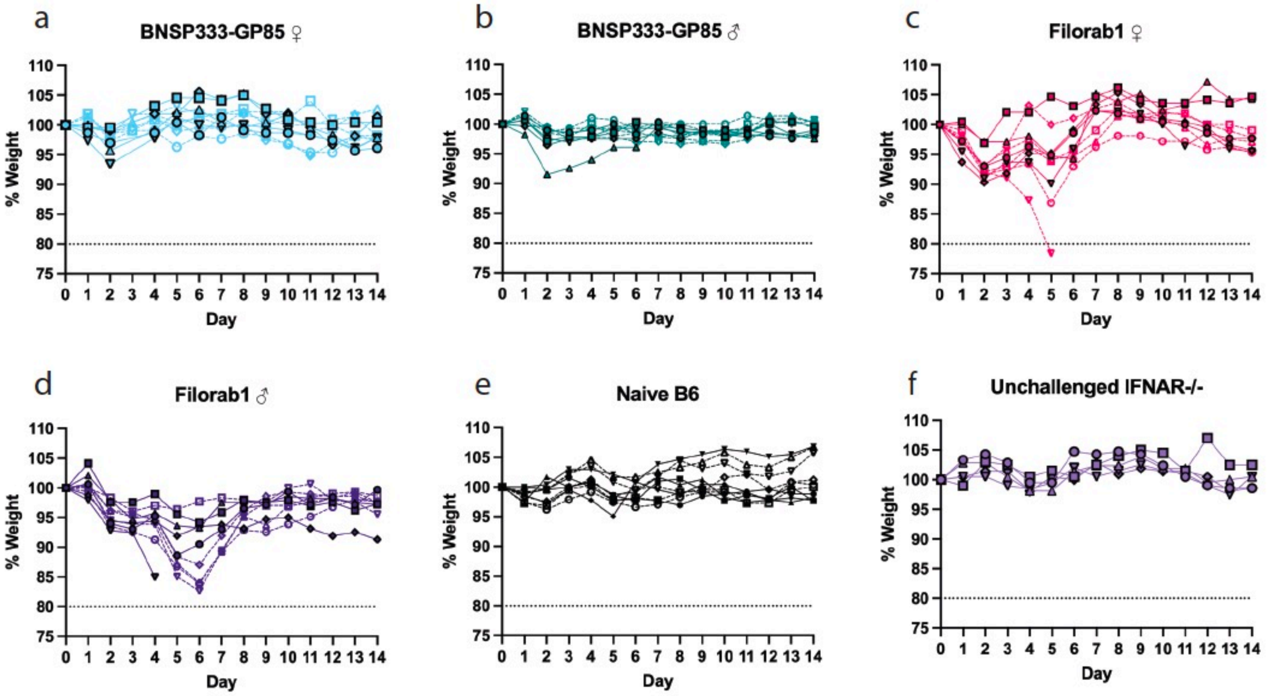

Supplemental Figure 6. Individual group weight curves of mice challenged with the surrogate challenge virus. Curves represent the percent change in weight from the day of challenge. Dotted line represents 20% weight loss, the point at which mice were euthanized. Results show the combination of two independent experiments; hollow symbols with a dotted connecting line represent the first experiment, and symbols with a black outline and solid connecting line represent the second experiment. Females from experiment two in panel A had their cage flooded on day 3, and thus the weights at this timepoint were excluded.

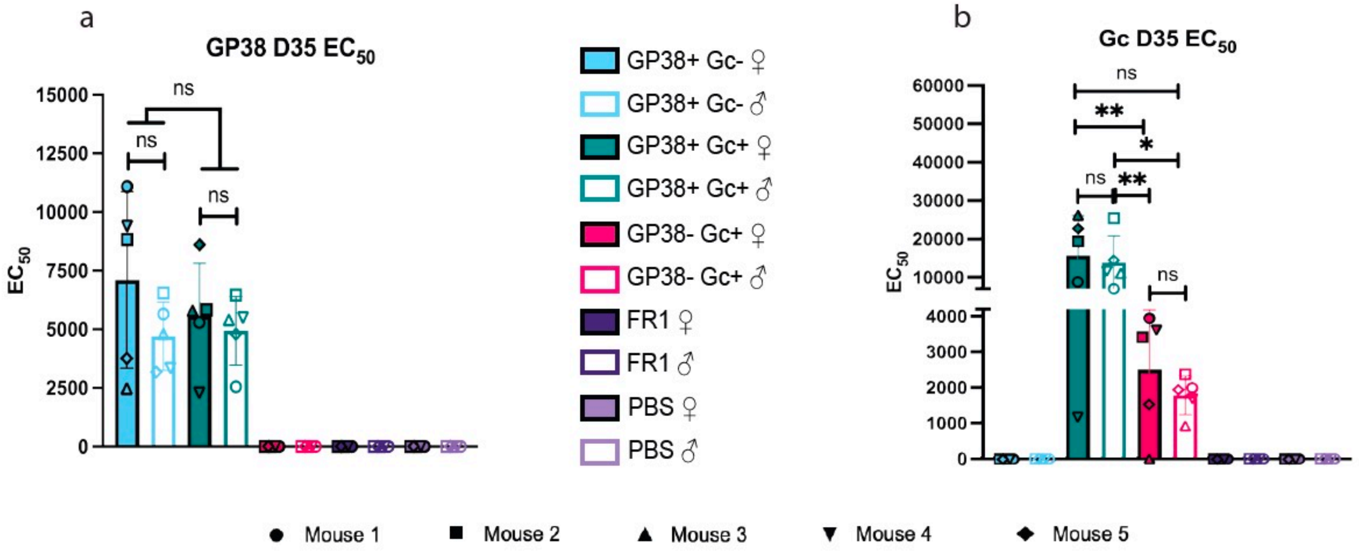

Supplemental Figure 7. Rhabdoviral-based CCHFV vaccines show no difference in immune responses between B6 males and females. Total IgG ELISAs against GP38 (A) or Gc (B) with sera from mice immunized for the CCHFV WT challenge experiment. Groups of 10 mice, 5 male and 5 female, were immunized with 10μg per dose of BPL inactivated vaccine adjuvanted with PHAD-SE. Error bars indicate the mean with standard deviation (SD) for groups of 5 mice with samples run in duplicate. An ordinary one-way ANOVA with Tukey's Multiple Comparison Test was used to determine statistical differences between groups at each time point. All groups with detectable antibody titers have 4-star significance compared to groups where no antibody titers were detected (****$P < 0.0001$; ***$P < 0.0002$; **$P < 0.0021$; *$P < 0.0332$; ns = not significant).

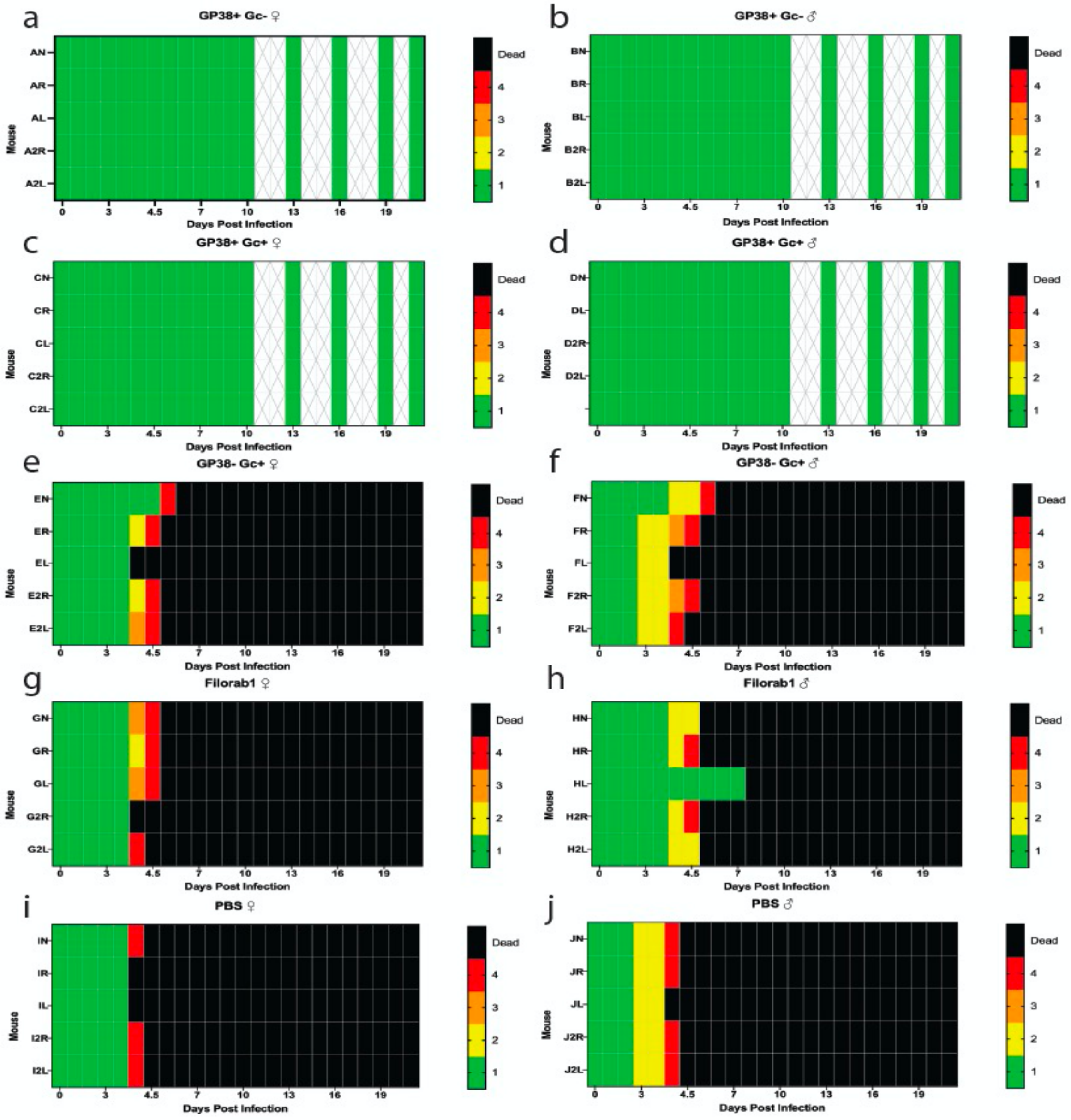

Clinical Scoring Criteria
**1** Healthy
**2** Ruffled fur, lethargic (triggers 2nd observation)
**3** Ruffled fur, lethargic, hunched posture, orbital tightening (Triggers 3rd observation)
**4** Reluctance to move when stimulated, paralysis, unable to access feed or water normally, moribund appearance, OR ≥20% weight loss<br>-- Immediate euthanasia --

Supplemental Figure 8. Clinical score heat maps from WT CCHFV challenge. Mice were given a clinical score from 1-4 that is represented by colors in the bars next to the heat maps. Each row represents an individual mouse, labeled based on their group and ear notches. Criteria for scores are listed in the table below the heat maps. Any time point where mice were not observed are crossed out with a gray X.

a

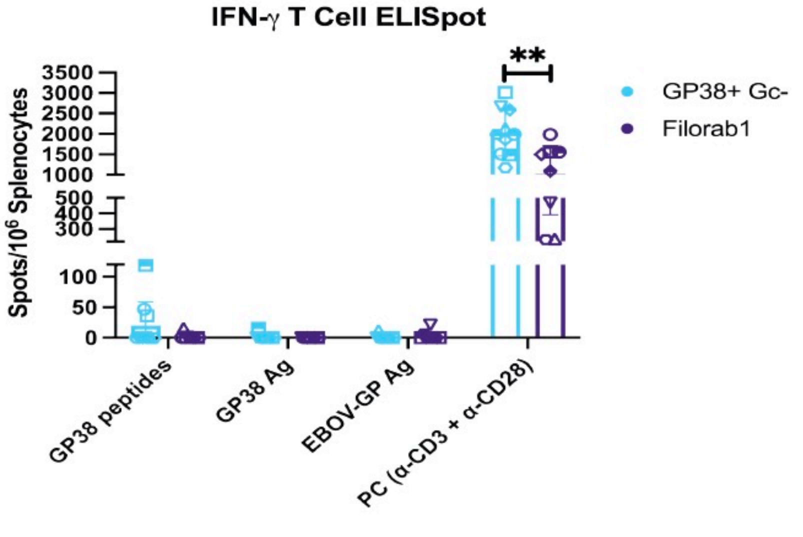

IFN-γ T Cell ELISpot
**
Spots/10^6 Splenocytes
3500
3000
2500
2000
1500
1000
500
400
300
100
50
0
GP38 peptides
GP38 Ag
EBOV-GP Ag
PC (α-CD3 + α-CD28)
GP38+ Gc-
Filorab1

b

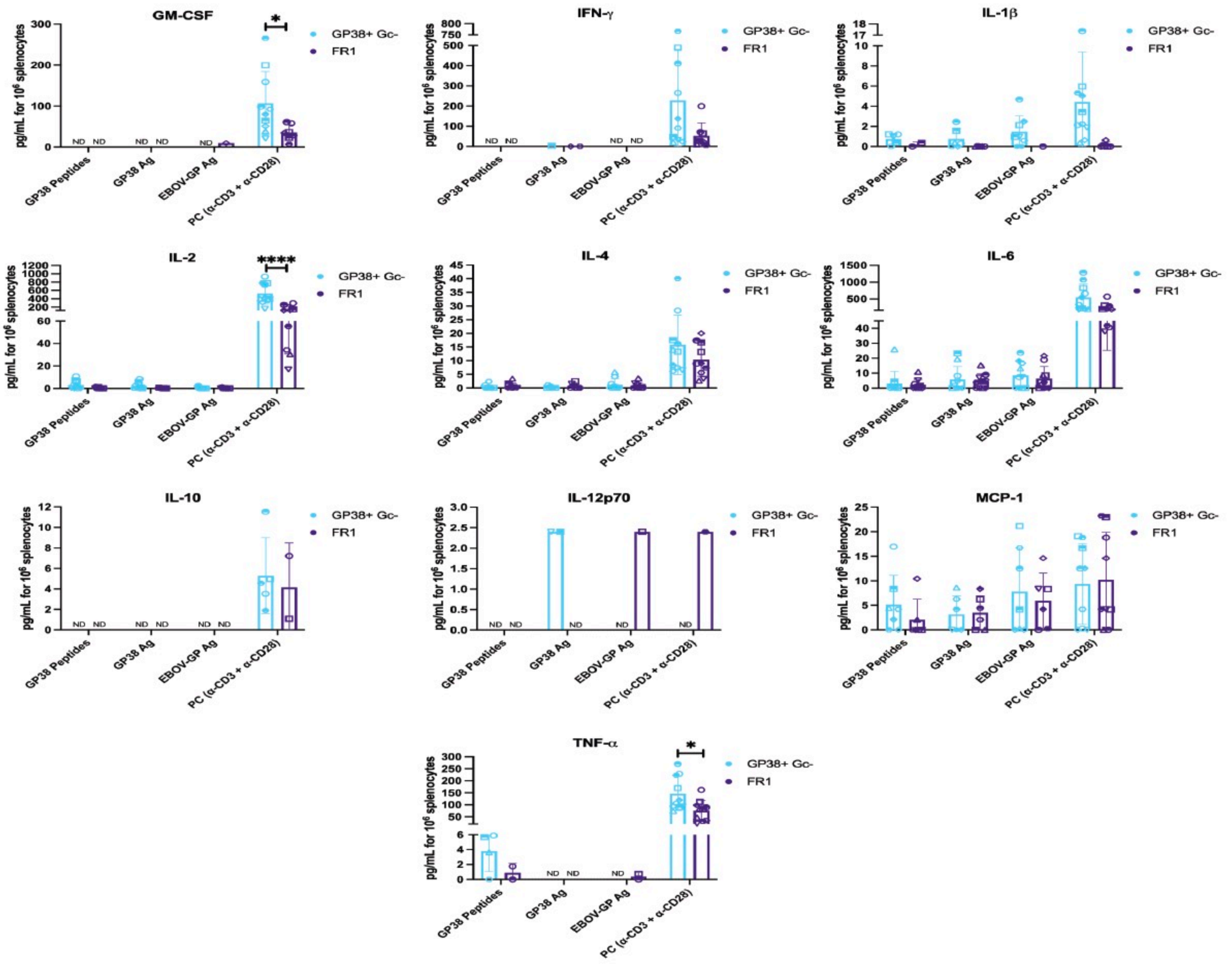

GM-CSF
*
GP38+ Gc-
FR1
pg/mL for 10^6 splenocytes
ND ND ND ND ND
GP38 Peptides
GP38 Ag
EBOV-GP Ag
PC (α-CD3 + α-CD28)
IFN-γ
ND ND ND ND
IL-1β
IL-2
****
IL-4
IL-6
IL-10
ND ND ND ND ND ND
IL-12p70
ND ND ND ND ND
MCP-1
TNF-α
*
ND ND ND

Supplemental Figure 9. GP38+ Gc- vaccine elicits minimal T cell responses. Groups of 10 mice were immunized with either GP38+ Gc- or FR1 and splenocytes were collected two weeks after the boost immunization. (A) Splenocytes were incubated with GP38 peptides, GP38 whole antigen (Ag), Ebolavirus glycoprotein (EBOV-GP) whole Ag or a positive control (PC) of α-CD3 and α-CD28 antibodies for 20hrs for an IFN-γ T cell ELISpot. Values were normalized for 10^6 cells and background corrected by subtracting the number of spots from unstimulated wells. (B) Supernatant from splenocytes incubated with the above stimulants was sent for cytokine multiplex analysis. Values were normalized for 10^6 cells and background corrected by subtracting the cytokine amounts from unstimulated wells. Each symbol represents an individual animal, and these symbols are kept consistent across the various panels in the figure. Error bars in both panels represent the mean with standard deviation. Statistical differences between vaccine groups for both assays were determined by the Mann Whitney test (****$P < 0.0001$; ***$P < 0.0002$; **$P < 0.0021$; *$P < 0.0332$; ns = not significant). ND, not detected.
