# Supplementary material for: GP38 as a vaccine target for Crimean-Congo hemorrhagic fever virus
Source: NPJ Vaccines. 2023 May 20;8:73. doi: 10.1038/s41541-023-00663-5 (PMC10199669; doi:10.1038/s41541-023-00663-5)
Supplement: Supplementary file 2 — REPORTING SUMMARY [file 41541_2023_663_MOESM2_ESM.pdf]

nature portfolio

Corresponding author(s): Dr Matthias Schnell

Last updated by author(s): Apr 3, 2023

Reporting Summary

Nature Portfolio wishes to improve the reproducibility of the work that we publish. This form provides structure for consistency and transparency in reporting. For further information on Nature Portfolio policies, see our Editorial Policies and the Editorial Policy Checklist.

Statistics

For all statistical analyses, confirm that the following items are present in the figure legend, table legend, main text, or Methods section.

n/a	Confirmed	
☐	☒	The exact sample size (*n*) for each experimental group/condition, given as a discrete number and unit of measurement
☐	☒	A statement on whether measurements were taken from distinct samples or whether the same sample was measured repeatedly
☐	☒	The statistical test(s) used AND whether they are one- or two-sided<br>*Only common tests should be described solely by name; describe more complex techniques in the Methods section.*
☒	☐	A description of all covariates tested
☐	☒	A description of any assumptions or corrections, such as tests of normality and adjustment for multiple comparisons
☐	☒	A full description of the statistical parameters including central tendency (e.g. means) or other basic estimates (e.g. regression coefficient) AND variation (e.g. standard deviation) or associated estimates of uncertainty (e.g. confidence intervals)
☒	☐	For null hypothesis testing, the test statistic (e.g. *F*, *t*, *r*) with confidence intervals, effect sizes, degrees of freedom and *P* value noted<br>*Give P values as exact values whenever suitable.*
☒	☐	For Bayesian analysis, information on the choice of priors and Markov chain Monte Carlo settings
☒	☐	For hierarchical and complex designs, identification of the appropriate level for tests and full reporting of outcomes
☒	☐	Estimates of effect sizes (e.g. Cohen's *d*, Pearson's *r*), indicating how they were calculated

Our web collection on statistics for biologists contains articles on many of the points above.

Software and code

Policy information about availability of computer code

Data collection	N/A
Data analysis	N/A

For manuscripts utilizing custom algorithms or software that are central to the research but not yet described in published literature, software must be made available to editors and reviewers. We strongly encourage code deposition in a community repository (e.g. GitHub). See the Nature Portfolio guidelines for submitting code & software for further information.

Data

Policy information about availability of data

All manuscripts must include a data availability statement. This statement should provide the following information, where applicable:

- Accession codes, unique identifiers, or web links for publicly available datasets
- A description of any restrictions on data availability
- For clinical datasets or third party data, please ensure that the statement adheres to our policy

All data necessary for evaluating the conclusions of this manuscript are included in the paper.

Human research participants

Policy information about studies involving human research participants and Sex and Gender in Research.

Reporting on sex and gender	N/A no human research participants
Population characteristics	N/A no human research participants
Recruitment	N/A no human research participants
Ethics oversight	N/A no human research participants

Note that full information on the approval of the study protocol must also be provided in the manuscript.

Field-specific reporting

Please select the one below that is the best fit for your research. If you are not sure, read the appropriate sections before making your selection.

☒ Life sciences ☐ Behavioural & social sciences ☐ Ecological, evolutionary & environmental sciences

For a reference copy of the document with all sections, see nature.com/documents/nr-reporting-summary-flat.pdf

Life sciences study design

All studies must disclose on these points even when the disclosure is negative.

Sample size	A group size of 5 mice was determined to be sufficient to observe statistically significant differences in the immune responses of mice receiving different vaccines and control groups.
Data exclusions	For the isotype ratios, any animals with undetectable IgG1 EC50 titers were excluded (Figure 4).<br>In the surrogate challenge model, the weight data on day 3 for the group of females receiving the GP38+ Gc- vaccine in the repeat of the experiment was excluded due to flooding in the cage that caused weight loss unrelated to the challenge (Figure 5d and S6). Additionally, any day 0 post challenge sample showing detectable viral RNA were considered contaminated and not reported.
Replication	Samples from groups of 5 mice were analyzed in either duplicate or triplicate. The surrogate challenge experiment was repeated a second time.
Randomization	The TJU laboratory animal services randomly assigned mice to the cages.
Blinding	None of the assays were performed blinded as the same researcher performed the immunizations, sample collections and ELISAs.

Reporting for specific materials, systems and methods

We require information from authors about some types of materials, experimental systems and methods used in many studies. Here, indicate whether each material, system or method listed is relevant to your study. If you are not sure if a list item applies to your research, read the appropriate section before selecting a response.

Materials & experimental systems

n/a	Involved in the study	
☐	☒	Antibodies
☐	☒	Eukaryotic cell lines
☒	☐	Palaeontology and archaeology
☐	☒	Animals and other organisms
☒	☐	Clinical data
☒	☐	Dual use research of concern

Methods

n/a	Involved in the study	
☒	☐	ChIP-seq
☐	☒	Flow cytometry
☒	☐	MRI-based neuroimaging

Antibodies

Antibodies used	Mouse monoclonal antibodies (mAb) anti-CCHFV-Gc (11E7, NR-40277) and anti-CCHFV-GP38 (13G8, NR-40294; 10E11, NR-40276) were obtained through the NIH Biodefense and Emerging Infections Research Resources Repository, NIAID, NIH. 4C12 human anti-

	RABV G mAb was produced from a 4C12 hybridoma (from Dr. Scott Dessain, Lankenau Institute for Medical Research, Wynnewood, PA). 1C5 mouse anti-RABV G mAb was purchased from Abcam® (Cat#Ab82460; RRID: AB_1658373). Hyperimmune mouse ascitic fluid (HMAF) was kindly provided by T. Ksiazek, University of Texas Medical Branch, Galveston, TX. Secondary antibodies Goat anti-mouse Alexa Fluor™ (AF) 568 (Invitrogen, Cat # A-11004) and Goat anti-human AF647 (Invitrogen, Cat # A48279) were purchased from ThermoFisher. Goat anti-mouse Brilliant Violet (BV) 510™ (Cat # 405331) was purchased from BioLegend®. Horseradish peroxidase (HRP)-conjugated goat anti-mouse IgG Fc-HRP (Cat # 1033-05) and Goat anti-human IgG-HRP (Cat # 2040-05) were purchased from SouthernBiotech. Goat anti-mouse IgG (H+L)-HRP (Cat # 115-035-146), goat anti-rabbit IgG (H+L)-HRP (Cat # 111-035-144) goat anti-mouse IgG, Fcgamma subclass 1 (Cat # 115-035-205), goat anti-mouse IgG, Fcgamma subclass 2b (Cat # 115-035-207), and goat anti-mouse IgG, Fcgamma subclass 2c (Cat # 11-035-208) were purchased from Jackson ImmunoResearch. Additionally, FITC anti-RABV N monoclonal globulin was purchased from Fujirebio® (catalogue number: 800-092).
Validation	These antibodies have been validated by testing them on appropriate controls (i.e. uninfected cells, vaccines not containing the target protein, etc.)

Eukaryotic cell lines

Policy information about cell lines and Sex and Gender in Research

Cell line source(s)	VERO-E6, ATCC; 239T, available from the Schnell laboratory; BSR cells, a BHK clone from the federal research center for viral diseases of animals, Germany; BEAS-2B, ATCC; 293F, ATCC; NA; available from the Schnell laboratory; Huh-7, available from the Bente laboratory; SW-13, provided by Dr. Éric Bergeron at the US Centers for Disease Control.
Authentication	Has not been performed
Mycoplasma contamination	All cell lines previously tested negative for mycoplasma contamination. Contamination is unlikely, however, cannot be completely excluded since it can occur every time cells are used.
Commonly misidentified lines (See ICLAC register)	N/A

Animals and other research organisms

Policy information about studies involving animals; ARRIVE guidelines recommended for reporting animal research, and Sex and Gender in Research

Laboratory animals	C57BL/6 mice, males and females; IFNAR-/- mice, males and females
Wild animals	N/A
Reporting on sex	For all challenge studies we tested both males and females. In the wildtype CCHFV challenge study, we saw no differences between sexes, but left these data separate to demonstrate the lack of differences. In the surrogate challenge model, we saw sex differences in both the EC50 antibody titers elicited by the vaccine between males and females before challenge, which correlated to differences in the disease progression between males and females in this model. This data has been kept separated.
Field-collected samples	N/A
Ethics oversight	IACUC, Thomas Jefferson University; IACUC, University of Texas Medical Branch

Note that full information on the approval of the study protocol must also be provided in the manuscript.

Flow Cytometry

Plots

Confirm that:

☒ The axis labels state the marker and fluorochrome used (e.g. CD4-FITC).

☒ The axis scales are clearly visible. Include numbers along axes only for bottom left plot of group (a 'group' is an analysis of identical markers).

☒ All plots are contour plots with outliers or pseudocolor plots.

☒ A numerical value for number of cells or percentage (with statistics) is provided.

Methodology

Sample preparation	VERO-E6 cells (ATCC) were infected with the various vaccine viruses, fixed with 2%pfa for 10 minutes and stained with the indicated antibodies to look at surface expression of viral antigens.
Instrument	BD FACSCelesta Flow Cytometer, 660344
Software	BD FACSDiva Software was used to collect data and FlowJo software (Treestar, Ashland, OR) was used to analyze the data.

Cell population abundance	Cells were infected at a high multiplicity to ensure that all were infected with the indicated vaccine virus.
Gating strategy	As all cells were infected, there was no need to gate out individual populations. All VeroE6 cells were gated and then the analysis was done using histograms of an individual fluorophore (i.e. antigen) to determine the geometric mean.

☒ Tick this box to confirm that a figure exemplifying the gating strategy is provided in the Supplementary Information.
